# Bio-Evaluation of ^99m^Tc-Labeled Homodimeric Chalcone Derivative as Amyloid-β-Targeting Probe

**DOI:** 10.3389/fmed.2022.813465

**Published:** 2022-06-17

**Authors:** Garima Mann, Kanchan Chauhan, Vikas Kumar, Shivani Daksh, Nikhil Kumar, M. Thirumal, Anupama Datta

**Affiliations:** ^1^Institute of Nuclear Medicine and Allied Sciences, Defence Research and Development Organization, New Delhi, India; ^2^Department of Chemistry, University of Delhi, New Delhi, India; ^3^Centro de Nanociencias y Nanotecnología, Universidad Nacional Autónoma de México, Ensenada, Mexico

**Keywords:** beta-amyloid, ^99m^Tc, SPECT, DTPA, chalcone

## Abstract

Chalcone derivatives have been successfully utilized for a range of biological applications and can cross the blood–brain barrier easily. β-amyloid-specific bis-chalcone derivative, 6,9-bis(carboxymethyl)-14-(4-[(E)-3-(4-(dimethylamino)phenyl)acryloyl]phenoxy)-3-(2-[(2-(4-[(E)-3-(4-(dimethylamino)phenyl)acryloyl]phenoxy)ethyl)amino]-2-oxoethyl)-11-oxo-3,6,9,12-tetraazatetradecanoic acid, DT(Ch)_2_, was analyzed using molecular modeling to explain the binding modes of the ligand with amyloid fibril and monomer followed by ^99m^Tc-complexation in 95% yield and 98.7% efficiency. High-binding specificity of the radiocomplex was established following *in vitro* evaluation against 100-fold excess of DT(Ch)_2_. ^99m^Tc–DT(Ch)_2_ exhibited <3% trans-complexation in human serum after 24 h, indicating high stability. A fast clearance rate in pharmacokinetics studies displayed a biphasic pattern with *t*_1/2_(*F*) = 30 min ± 0.09 and *t*_1/2_(*S*) = 4 h 20 min ± 0.06. *In vivo* single-photon emission computed tomography (SPECT) imaging in rabbits reiterated the pharmacokinetics data with initially high brain uptake followed by rapid washout. Biodistribution studies confirmed the initial brain uptake as 1.16 ± 0.02% ID/g after 2 min and the brain_2min_/brain_30min_ ratio was 3.74. Radioactivity distribution in the brain was >40% in the cingulate cortex followed by >25% in the hippocampus, a distribution pattern aligned to Alzheimer’s affected brain regions. Radiocomplex also displayed rapid plasma clearance followed by hepatobolic and renal modes of excretion.

## Introduction

A substantial proportion of current and past literature has focused on utilizing amyloid plaque formation as the focal point to understand the etiology and pathogenesis leading to Alzheimer’s Disease (AD) and similar psychoneurological disorders (1, 2). The amyloid cascade hypothesis suggests sequential proteolytic processing of amyloid precursor protein (APP) by BACE (β-secretase) and γ-secretase into various smaller fragments of varying peptide lengths, of which Aβ_40_ and deleterious Aβ_42_ are the most common forms causing the formation of senile plaques in the human brain (3, 4). The Aβ assemblies encompass monomers, oligomers, and fibrils with varying aggregation susceptibility, among which oligomers have been associated with synaptic toxicity and plaques that are linked to the pro-inflammatory effects (4, 5). These different plaque forms together have a propensity for neuronal toxicity (6). There is mounting evidence also stipulating a neuropathological link between traumatic brain injury (TBI) and early onset of AD (7, 8). The existence of amyloid plaques has been observed immediately after the incidence of TBI in the patient’s brains as well as in various animal models (9, 10). The early-stage identification of these Aβ assemblies is therefore indispensable for the pre-symptomatic identification of patients at the risk of cognitive deficits and for understanding the molecular mechanistics of the relationship between TBI and AD (11).

The universality of the amyloid cascade hypothesis has led to these plaques being most explicitly targeted by various imaging techniques. Such techniques include, but are not limited to, optical imaging, single photon emission computed tomography (SPECT), magnetic resonance imaging (MRI), and positron emission tomography (PET) in reported literature as well as in clinical diagnosis (12–15). SPECT imaging is a widely applicable yet economical alternative to PET imaging without considerable loss of sensitivity and spatial resolution. ^99m^Tc, a SPECT radiometal, has remained a cornerstone in the field of diagnostic nuclear medicine owing to it being readily available as a commercial generator. Its various favorable nuclear properties such as the half-life of 6 h, pure gamma-ray energy (*E* = 140 keV), and reduced radiation burden make it ideal for imaging applications (16).

**FIGURE 1:**
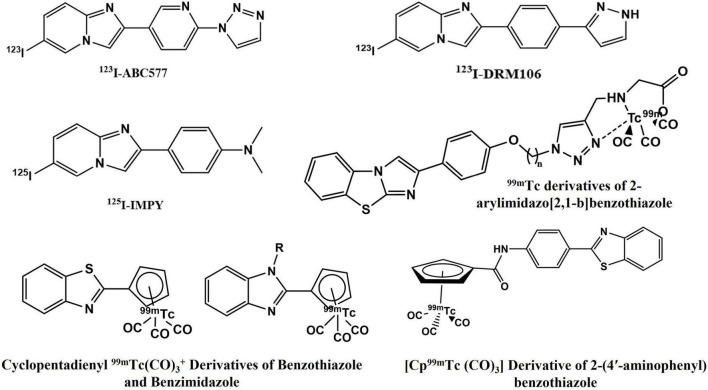
Aβ_42_-specific single photon emission computed tomography (SPECT) imaging probes.

Suitable cost-effective SPECT agents like several small and neutral ^99m^Tc-labeled derivatives of chalcone, flavone, aurone, curcumin, benzothiazole, aryl benzoxazole, aryl benzofuran, avone, and dibenzylideneacetone have demonstrated high Aβ-binding affinity and preferential brain uptake (17–22). [^123/125^I]DRM106, [^125^I]IMPY, and [^123^I]ABC577 are some of the SPECT probes under clinical investigation (Figure 1; 4, 23, 24). Despite these advances, the amyloid-specific SPECT imaging probe development has been slow and none has yet been clinically approved.

**FIGURE 2:**
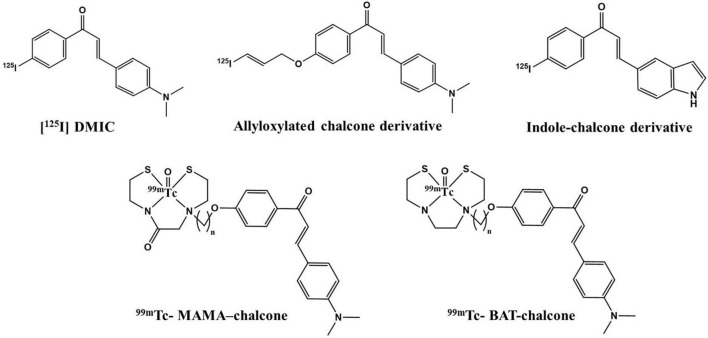
Chalcone derivatives as Aβ_42_-specific single photon emission computed tomography (SPECT) imaging probes.

Recently, chalcone derived from naturally occurring flavonoids and its analog have gained significant attention in developing AD-specific imaging agents. Its easy synthesis and convenience in modification of lipophilicity (log P) led researchers to develop various chalcone-based SPECT agents for Aβ imaging (Figure 2). Besides, this scaffold is privileged with different medicinal applications and its derivatives are already in clinical practice as choleretic, vascular protective, mucoprotective, and antiulcer drugs (25). Previously developed ^68^Ga-labeled homodimeric chalcone–DTPA complex displayed promising results in preliminary PET imaging studies (26). Increased specificity due to the presence of *N*,*N*-dimethylamino groups on the chalcone moiety and thermodynamically stable dimeric structure make this molecule a potential candidate for utilization as an imaging probe (27–29).

Simultaneously, diethylenetriaminepentaacetic acid (DTPA) is largely exploited as a chelating agent due to its ease and efficiency of binding to various radionuclides such as ^99m^Tc and ^111^I in high yield and purity (30, 31). However, the requirement of the on-site cyclotron and high economic burden largely limits the routine utilization of PET imaging. In this direction, the incorporation of the above scaffold as a cost-effective SPECT imaging ligand provides a feasible alternative to the PET modality. ^99m^Tc is the radionuclide of choice due to its rich chelating chemistry and low-radiation burden. The scaffold was complexed with ^99m^Tc and studied for its efficacy as a SPECT imaging radiotracer. Molecular docking studies performed on the ligand aid in understanding the interactions with monomeric and fibrillar protein forms. The radiolabeling parameters were optimized and the *in vitro* stability of the radioligand was evaluated followed by *in vivo* gamma scintigraphy and biodistribution studies to establish its suitability for *in vivo* imaging.

## Experimental Section

### Chemicals

Reagent grade chemicals purchased from Merck, Germany were used without additional purification steps unless otherwise specified. Thin-layer chromatography (TLC; Silica gel 60, F_254_, Merck) was used to monitor the reactions and silica MN60 (60–200 μm) was used for open-column chromatography. ^99m^Tc was procured from the Regional Center for Radiopharmaceuticals (Northern Region), Board of Radiation and Isotope Technology (BRIT), Department of Atomic Energy, India. HEK-293 (Human Embryonic Kidney 293) cells were procured from the National Center for Cell Science, Pune, India.

### Instrumentation

The ^1^H (400 MHz) and ^13^C (100 MHz) NMR spectra were performed using a Bruker Avance II spectrometer. The mass spectra were obtained on an Agilent 6310 ion trap mass spectrometer. A UV detector (λ = 214 and 254 nm) coupled with Agilent 1200 LC was used for the HPLC analysis using a C-18 RP Agilent column (5 μm) with dimensions of 4.6 mm × 250 mm. The mobile phase consisted of a mixture of water (A) and acetonitrile (B) with 0.1% trifluoroacetic acid and a gradient elution technique was followed for separation. The flow rate ranged between 0.5 and 6 mL/min for analytical and preparative HPLC, respectively. The Schrodinger software was employed for computational studies. An ITLC-SG (Gelman Science, NY, United States) was used to check the radiochemical purity and radiocomplexation, while the γ-scintillation counter GRS230, ECIL (Hyderabad, India) was used for the labeling studies. The SPECT imaging was performed on a γ-camera Hawkeye Camera (WI, United States).

### Animal Models

Scintigraphy and blood clearance studies were carried out on New Zealand rabbits (2–3 kg), while normal BALB/c mice (male, 5–6 weeks, and 25–30 g) were sacrificed for biodistribution studies. The approval for animal experiments was acquired by the Institutional Animals Ethics Committee. The rabbits were housed in individual cages while mice were placed two per cage in rooms maintained at 21 ± 3°C with a 12 h light/dark cycle. There was no restriction on the access to food and water.

### Molecular Modeling Studies

#### Ligand Preparation

Ligand empirical structures (drawn using CHEM DRAW 12th VERSION) were converted to the MOL-SD files. After importing the MOL file into the Maestro workspace, the Ligprep [a Schrodinger software suite used for generating 3D conversion of 1D (Smiles) and 2D (SDF) structures] was used for generating the ligand structures. After defining the correct atom and bond types, assigning protein charges according to the force field method, and adding hydrogens, the GLIDE force field performed a short-energy minimization to release the internal strain. The ionizable amino groups were then subsequently protonated to satisfy the biological pH (29).

#### Protein Preparation

The Schrodinger protein preparation wizard tool is used for preparing and refining the PDB structures of 1IYT (monomer) and 2BEG (fibrils). This was done by assigning bond orders, adding hydrogens, correcting charges, optimizing hydrogen bonds, and finally minimizing the protein structure. The side chains that were not in proximity of the binding cavity along with those not participating in salt bridges were neutralized with subsequently limited minimization of the co-crystallized complex. The result is mitigation of potential steric clashes and reorientation of side-chain hydroxyl groups. An OPLS_2005 force field with a Polack-Ribiere Conjugate Gradient (PRCG) algorithm was used for the minimization of ligand, which was stopped following either convergence of energy gradient <0.05 kCal/mol or completion of 5,000 steps. For structures lacking any side-chain atoms, conformations were predicted using the refinement module in the Prime (Prime, v2.0, Schrodinger, LLC, New York, NY, United States). To alleviate pre-existing steric clashes in the original PDB protein structures, an all-atom constrained minimization was performed for a brief relaxation using the Impact Refinement module (Impref) (Impact v5.0, Schrodinger, LLC, New York, NY, United States) using the OPLS-2005 force field. The minimization termination occurred when RMSD reached 0.30 Å cutoff or the energy converged. The final receptor grid was generated after adding hydrogen atoms and partial charges.

#### Docking Methodology

Docking experiments were performed on the IFD (induced fit docking) program (28) from the extra precision (XP) mode calculations. The prepared protein and ligand were put together to initiate the docking *via* flexible docking mode. The auto-generated conformations pass through the filters to gauge the ligand–receptor interactions. The primary filters tested the ligand active site spatial fit, and their interaction complementarities by grid-based pattern following the empirical chemscore function. Following initial screenings, grid approximation was minimized and poses were evaluated according to the OPLS-AA non-bonded ligand–receptor interaction energy. The analysis of the binding affinity and final scoring on the energy minimized poses and results were performed using the Schrodinger’s proprietary glide score multi-ligand scoring function.

### Radiolabeling Studies

Radiolabeling with ^99m^Tc was performed as described previously (16). DT(Ch)_2_ (5.0 μmol) was dissolved in saline (PBS pH 7.4, 100 μL) in a shielded vial. To this solution, an appropriate concentration of stannous chloride dissolved in 10% CH_3_COOH (N_2_ purged) was added, followed by pH adjustment to seven using 0.5 M NaHCO_3_. Filtration *via* 0.22 μm syringe filters was followed by the addition of freshly eluted (<1 h) 3.7 MBq of Na^99m^TcO_4_ and 10 min incubation at room temperature. The labeling efficiency and purity of the complex formed were checked on the ITLC-SG strips by ascending instant TLC. For the mobile phase, two systems were tested: pyridine/acetic acid/water (PAW) 3:5:1.5 and 100% acetone, and the results were used for the calculation of labeling efficiency. ^99m^Tc-complex purification was performed by a C-18 reversed-phase extraction cartridge. The profile of the purified ^99m^Tc–DT(Ch)_2_ on the TLC strip was scanned in the TLC scanner and the purity was established using a radio HPLC using a mixture of ACN/H_2_O (6:4) as a mobile phase with the flow rate of 4 mL/min.

### Partition Coefficient Determination

The partition coefficient of complex ^99m^Tc–DT(Ch)**_2_** was determined using the same general procedure (26). In the equal volume suspension of 0.1 M phosphate buffer (pH 7.4) and *n*-octanol, radiotracer was added and followed by 3 min vortex and subsequent centrifugation (5,000 rpm) for 5 min. Equal volumes from organic and inorganic layers were separately measured in a well-type gamma counter. The final partition coefficient (log *P*) was calculated by the logarithmic ratio of count per gram of *n*-octanol PBS. The measurements were done in triplicates.

### *In vitro* Binding and Blocking Assay on Aβ_1–42_ Aggregates

The peptide fragment of Aβ_42_ (Sigma-Aldrich) was aggregated by dispersing the peptide in sodium phosphate (10 mM) and EDTA (1 mM) buffer solution (pH 7.4) by incubating for 42 h at 37°C. The binding specificity to Aβ plaques of ^99m^Tc–DT(Ch)_2_ was assessed by binding assay. A total of 100 μL aggregated Aβ_42_ (2 μM in final concentration) with 100 μL ^99m^Tc–DT(Ch)_2_ and 900 μL PBS was mixed in borosilicate glass tubes. Saturation studies were performed using 0.5–100 nM ^99m^Tc–DT(Ch)_2_ mixed with a non-labeled compound. For inhibition studies, 29 nM Aβ plaques (pre-aggregated, 100 μL), 0.10 nM ^99m^Tc–DT(Ch)_2_ (100 μL), 10^–5^–10^–10^ M inhibitors (in 5% EtOH, 100 μL), and 0.2 M of PBS (700 μL, pH = 7.4) were mixed. DT(Ch)_2_ (400 nM) was used for defining a non-specific binding. During the blocking assay, 100-fold excess unlabeled DT(Ch)_2_ and DMIC moieties were used for the estimation of non-specific binding. Following 2 h incubation at 37°C, GF/B Whatman filters were used to segregate free and bound radioactivity, which was measured using a gamma scintillation counter. Specific binding was obtained as the difference of the total from the non-specific binding.

### Human Serum Stability Studies

Radiotracer stability was tested in human serum. Freshly extracted human blood serum (500 μL) was incubated with 200 μL of the radiotracer and its dissociation was analyzed from 0 to 24 h *via* ITLC and the developing solvent was pyridine/acetic acid/water (3:5:1.5).

### Blood Kinetics Evaluation

^99m^Tc–DT(Ch)_2_ (200 μL, 12 MBq) was injected *via* the dorsal ear vein of normal rabbits. Blood was withdrawn at pre-determined intervals between 5 min and 24 h from the ear vein. The activity persistence was calculated by approximating the total blood volume as 7% w.r.t. the body weight.

### Cytotoxicity Assay

*In vitro* cytotoxicity studies were performed for DT(Ch)_2_ in the concentration range of 0.5–50 μg/mL against HEK 293 cell lines seeded in 96-well plates. The experimental procedure followed was in accordance with the previously reported literature (32). An MTT assay was employed, and the cell viability was observed for up to 48 h. Cell cultures were grown in DMEM (Dulbecco’s modified eagle medium, high glucose) and the culture was supplemented with 1% penicillin streptomycin and 10% FBS (fetal bovine serum). The cells were incubated with different compound concentrations in a 5% CO_2_ incubator at 37°C. Following the addition of MTT [3-(4,5-dimethylthiazol-2-yl)-2,5-diphenyl-2H-tetrazolium bromide] to each cell (100 μL), the systems were incubated for an additional 4 h. The cell viability was reported in accordance with the mitochondrial activity measured *via* absorbance at 570 nm. The experiments were performed in triplicates.

### Hemolysis Assay

In accordance with the previous literature, heparinized blood from healthy volunteers was used for the hemolysis assay (33). Blood serum was separated by centrifugation and the obtained RBC pellet was washed with PBS (pH = 7.4) repeatedly. The pellet was finally resuspended in PBS and added in equal amounts to different vials. Similarly, varying concentrations of the ligand in equal volume were incubated with the RBC pellet in the ratio of 8:2 at 37°C. The Triton X-100 and phosphate buffer saline as positive and negative control, respectively, were also incubated with RBCs under similar conditions. A total of 100 μL of solvent was centrifuged and removed at each time point: 15 min, 1, 2, 4, and 24 h. The absorbance of the supernatant was measured at 541 nm wavelength in a 96-well plate by a Synergy 2 Multi-Mode Reader (BioTek Instruments, United States). The experiments were carried out in triplicates and the extent of hemolysis was calculated according to the following formula:

$$\mathrm{Hemolysis}\,\mathrm{ratio}=\frac{\begin{array}{c} (\lambda \left(\mathrm{test}\right)\\ -\lambda \left(\mathrm{negative}\mathrm{control}\right)) \end{array}}{\begin{array}{c} (\lambda \left(\mathrm{positive}\mathrm{control}\right)\\ -\lambda \left(\mathrm{negative}\mathrm{control}\right)) \end{array}}\times 100\%$$

### *Ex vivo* Brain Section Staining

To evaluate the amyloid-binding affinity, an amyloid-overexpressing rodent (C57BL/6 mouse) model was used (34). The rodent was sacrificed, and the brain sections were fixed in formalin. The presence of amyloid plaques in the rodent model was confirmed through staining with dyes, Congo Red and Thioflavin S.

### *In vivo* Scintigraphy Studies

The SPECT imaging was performed on normal rabbits. Following anesthetization with diazepam, they were administered with 17.5 MBq of ^99m^Tc–DT(Ch)_2_ (100 μL) *via* the dorsal ear vein intravenously. The SPECT imaging was carried out for 20 min and a semiquantitative ROI analysis was performed.

### Biodistribution Studies

The intravenous administration of 100 μL^99m^Tc–DT(Ch)_2_ (∼1.5 MBq) *via* the tail vein in normal BALB/c mice and amyloid-overexpressing C57BL/6 mice was carried out, who were then sacrificed by cardiac puncture at pre-determined time intervals. Each organ of interest was washed with chilled saline, and radioactivity and weight were measured using a gamma-ray counter. The results were reported as the percentage injected dose per gram of the organ (% ID/g).

## Results and Discussion

The risk of developing various neurodegenerative disorders is one of the most profound and disabling ramifications of TBI. Pre-symptomatic identification of deleterious plaques is, therefore, imperative to regulate the condition and slow down the deterioration process. In this direction, SPECT imaging is an excellent non-invasive technique to identify amyloid plaques before the neuron degradation process initiates.

Polyaminopolycarboxylic ligands are constituted of multivalent functional groups rendering excellent suitability for conjugation to one or more biovectors and radionuclide simultaneously. DTPA is the ligand of choice owing to its compatibility with a wide array of both diagnostic and therapeutic radionuclides (35). Chalcone moiety has the advantages of facile synthesis, easy blood–brain barrier (BBB) penetration, and shows selectivity toward amyloid plaques. The work presented here follows an easy and high-yielding radioligand synthesis. Molecular modeling experiments were performed to evaluate the interaction of the ligand with different forms of amyloid-beta and assess its specificity. DT(Ch)_2_ was complexed with ^99m^Tc in high yield and purity to form a stable SPECT imaging probe, which was further evaluated for its biological activity and efficacy in crossing BBB to bind the deleterious Aβ_1–42_ plaques.

### Molecular Modeling Experiments

**FIGURE 3:**
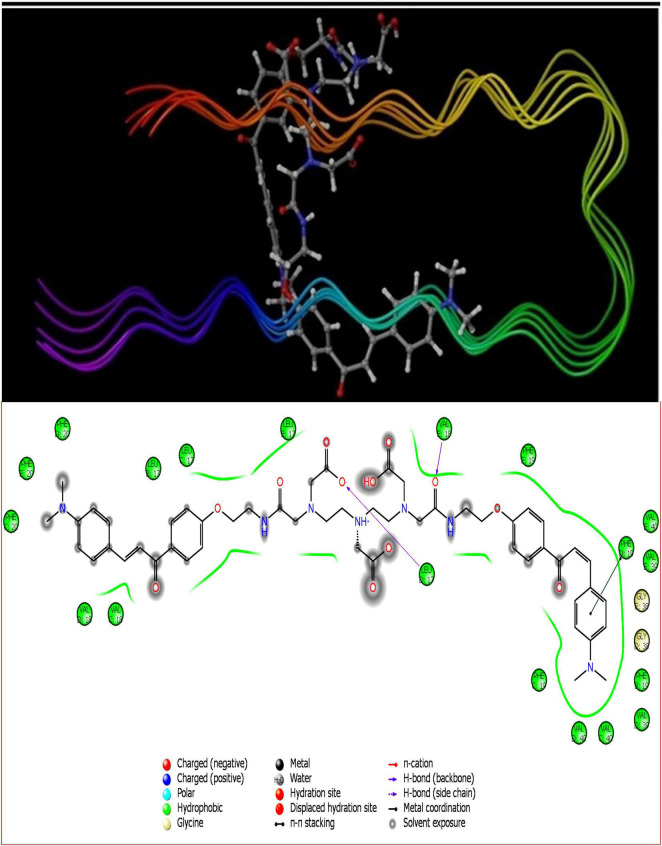
DT(Ch)_2_ 3D docking pose depicting the 2D and binding view with amyloid-beta fibril (2BEG).

**FIGURE 4:**
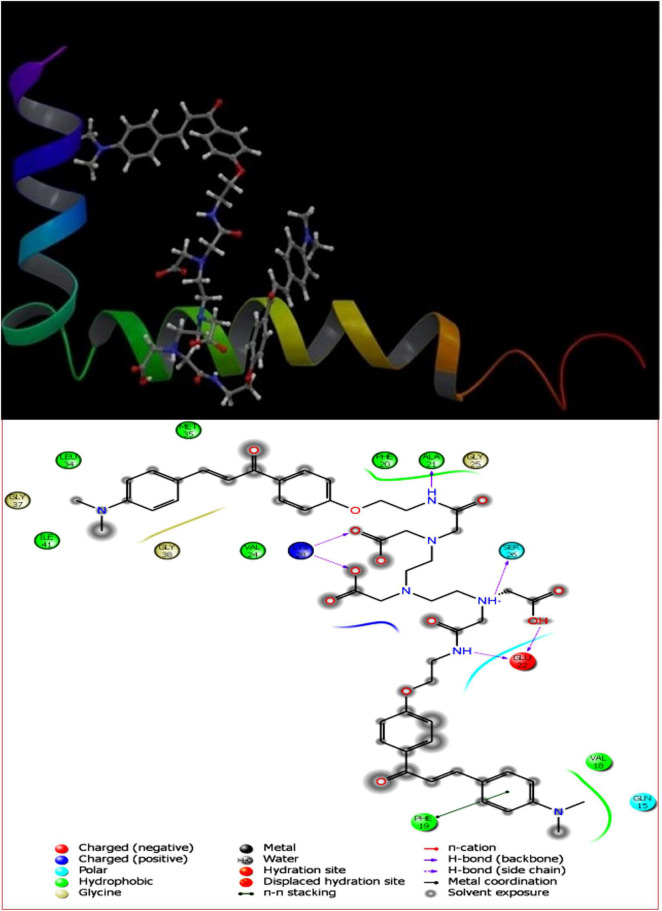
DT(Ch)_2_3D docking pose depicting the 2D and binding view of amyloid-beta monomer (1IYT).

The pivotal role of flavone derivatives, especially the chalcones, prompted us to conjugate it with the DTPA and to study the binding affinity *via* molecular modeling. The 3D and 2D diagrams were generated for ligand interaction with amyloid fibril and monomer (Figures 3, 4).

**FIGURE 5:**
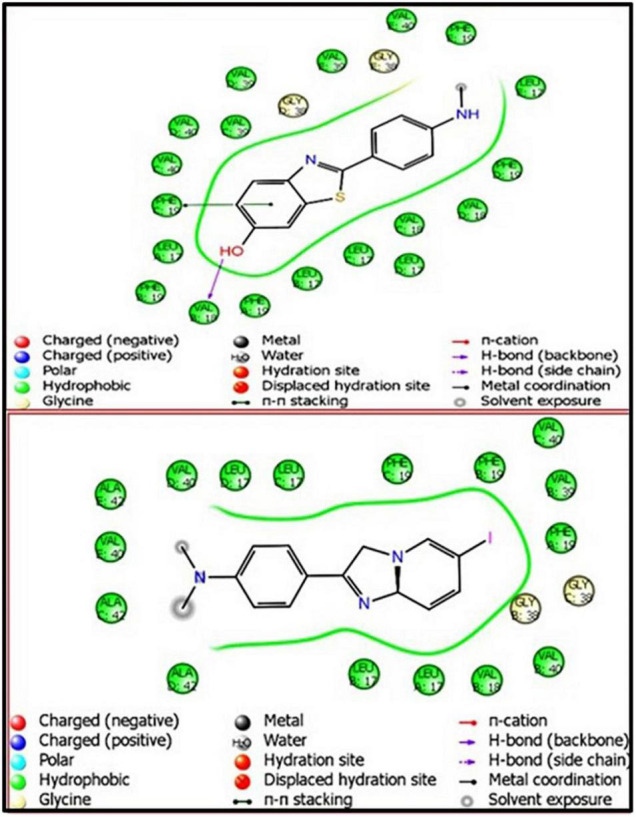
Ligand interaction diagram (LID) of PiB- and IMPY-associated amyloid-beta fibril (2BEG), respectively.

To account for the backbone and side-chain movements of the various receptor proteins during ligand binding and alleviate the misleading results that might arise from the assumption of the rigid receptor, the “induced-fit” approach has been followed in this study. The reason for the inclusiveness of these side-chain movements is to allow alteration in the binding site of the receptor so that it accommodates the binding mode and shape of the ligand. Docking studies of the DTPA conjugate of chalcone [DT(Ch)_2_] on the potential binding sites of the amyloid protein revealed their interaction modes, as well as structural and positional pre-requisites for potential amyloid fibril binding. We first docked to the X-ray crystallographic structure of the Aβ_42_ monomer (PDB ID: 1IYT) and found that Ala21, Glu22, Phe19, Ser26, and Lys28 were the main anchoring residues of amyloid monomer binding with the compound. In the docking simulation, the dimensions of the grids were 3.4944 × −1.5161 × −5.3684 and −14.3191 × 0.9368 × −11.9627 for monomer and amyloid fibril, respectively. It was evident from the docking results that our compound has a higher affinity (in terms of higher negative Glide GScore value) with amyloid fibril (2BEG) than their counterpart monomer protein (1IYT) (Table 1). As high-negative thermodynamic parameters suggest a strong binding affinity with the receptor, it ultimately justifies the utility of our compound for amyloid fibril in tandem. To comprehend interactions of the amyloid protein and compound, the receptor–ligand docking pose was generated. According to the two-dimensional ligand interaction diagram, the compound binds at multiple anchoring residues of different polypeptide chains present in amyloid fibril. For instance, amino acid Val17, Phe19 of sequence D, and Val18 of sequence E in amyloid fibril bind with the compound. However, only Phe19 showed the π–π interaction with the chalcone moiety of the compound. To carry out the comparative analysis of the docking scores of ligands, we have taken two known clinically employed molecules for the diagnosis of Aβ (Pittsburgh compound B, PiB, and 6-iodo-2-(4′-dimethylamino-)phenyl-imidazo[1,2-a]pyridine, IMPY) and used their receptor docking data as a reference interaction (Figure 5), which suggested the better binding affinity of our compound in comparison to the reference molecules (Table 1). The docking score of DT(Ch)_2_ was found higher than the currently FDA-approved tracers, such as florbetaben, florbetapir, and flutemetamol (36). Additionally, in comparison to previously reported chalcone derivatives, such as dihydrochalcone and Chal-Me, which have binding affinity to amyloid fibrils, binding energy for DT(Ch)_2_ was comparable but lower than the dimeric derivative of Chal-Me, (Chal)_2_DEA-Me ligand (32, 37). A similar range of binding energy was also reported for recently synthesized isoxazolone and triazole derivatives (38, 39).

**TABLE 1:** Thermodynamic parameters of induced-fit docking.

	Glide GScore	Glide evdW	Glide ecoul	Glide energy	Glide Hbond	IFD score
DT(Ch)_2_ with monomer (1IYT)	–6.132	–40.440	–27.233	–67.673	–2.966	–92.181
DT(Ch)_2_ with fibril (2BEG)	–8.881	–54.404	–5.505	–59.909	–0.928	–234.366
PiB with 2BEG	–6.190	–24.926	–0.883	–25.809	–0.318	–227.530
IMPY with 2BEG	–7.230	–36.132	–0.235	–36.367	–0.053	–224.088

Shape complementarity of the active site of amyloid protein and ligand is necessary for the protein–ligand complex interaction. Therefore, the results also highlight various ligand–protein atoms interactions like lipophilic, aromatic, polar, polar-lipophilic, and steric interactions, in addition to ligand acceptor–enzyme donor interactions, phenyl–amides/methyl/aryl CH interactions, and hydrogen bond interactions. The results are also inclusive of the frozen rotatable bond penalty and the penalty for ligand–enzyme clashes.

### Synthesis and Radiolabeling Studies With ^99m^Tc

Synthesis follows a bivalent approach and is a Claisen–Schmidt condensation yielding (E)-1-(4-hydroxyphenyl)-3-(4-isopropylphenyl)prop-2-en-1-one, which is further conjugated with DTPA to give 6,9-bis(carboxymethyl)-14-(4-[(E)-3-(4-(dimethylamino)phenyl)acryloyl]phenoxy)-3-(2-[(2-(4-[(E)-3-(4-(dimethylamino)phenyl)acryloyl]phenoxy)ethyl)amino]-2-oxoethyl)-11-oxo-3,6,9,12-tetraazatetradecanoic acid, DT(Ch)_2_ in 95% yield (26; Supplementary Figure 1).

DT(Ch)_2_ was labeled using ^99m^Tc for application in the SPECT imaging at pH 6.0–7.5 by incubating the reaction mixture at room temperature for 10 min. The complex formed was purified by using a pre-conditioned C-18 cartridge. The amount of stannous chloride used in the radiolabeling is crucial to achieve optimum labeling efficiency and to avoid the formation of radiocolloids (reduced/hydrolyzed ^99m^TcO_2_) or inferior labeling. The radiolabeling efficiency was 98.7% as estimated by ITLC-SG by 100% acetone, and PAW (3:5:1.5) as developing solvents. The radiochemical purity of the bioconjugate was 97.23% as determined using HPLC with retention time = 6.6 min (Supplementary Figure 2).

### Partition Coefficient Determination

BBB consists of tight junctions between endothelial cells, which discourage brain permeability by molecules. In living organisms, lipophilicity affects the distribution process along with other pharmacokinetic processes. It is a common descriptor of BBB permeability along with low-molecular weight (<600 Da) and charge neutrality. The log *P*-value helps to determine lipophilicity and values in the range of 1.0–3.5 facilitates high brain uptake and reduces non-specific binding. The log *P*-value of DT(Ch)_2_ was calculated to be 0.96 ± 0.06 (*n* = 5) at 7.4 PH, while complexation with ^99m^Tc led to a significant increase in lipophilicity, and a shift in log *P*-value to 1.76 ± 0.04 (*n* = 5, *p = 0.0035) at pH 7.4. Although both molecules have log *P*-values in the range facilitating BBB permeability, the value for ^99m^Tc–DT(Ch)_2_ was found to be higher than the corresponding ^68^Ga complex (1.58 ± 0.09), indicating higher lipophilicity of the ^99m^Tc complex. The increased values can be attributed to a more stable ^99m^Tc–DTPA complex resulting in a new pharmacological profile. Similar results were also reported for ^99m^Tc-PSMA-11 (40) and ^68^Ga-PSMA-11 (41), wherein the log *P*-value for ^99m^Tc complex displayed higher lipophilic property as compared to ^68^Ga complex.

### *In vitro* Binding and Blocking Assay Using Aβ_1–42_ Aggregates

**FIGURE 6:**
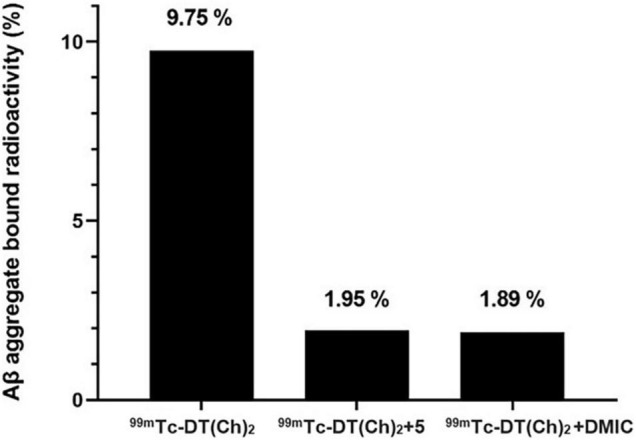
Binding and blocking assay of ^99m^Tc–DT(Ch)_2_ with Aβ42-plaques (*n* = 5). The binding is represented as %radioactivity bound to the Aβ42 aggregates.

A preliminary study for an Aβ(1–42)-targeting moiety is screening the molecule for its affinity toward the corresponding protein. For this, *in vitro* binding studies were employed using pre-aggregated forms of Aβ_42_ peptide. Aggregate-bound radioactivity (%) of the ^99m^Tc complex was analyzed and found to be ∼9.7% (Figure 6). The value was comparable to the previously reported chalcone mimicking ^99m^Tc cyclopentadienyl tricarbonyl complex (17). Saturation binding of ^99m^Tc–DT(Ch)_2_ to artificially aggregated Aβ_42_ suggested high affinity and one-site binding with *K*_*d*_ of 3.71 ± 0.38 nM (Supplementary Figure 4). The *K*_*d*_ value was within the range of ^125^I-DMIC (*K*_*d*_ = 4.2 nM), which is a well-known chalcone derivative (42). These values can be attributed to the existence of dimeric chalcone moiety as well as *N*,*N*-dimethylamino groups present on the chalcone scaffold (43). The size of the chelator also affects the binding affinity of the molecule. For instance, in a series of ^99m^Tc-labeled phenylquinoxaline derivatives, the presence of bis(aminoethanethiol) (BAT) as a chelating ligand decreased the binding affinity (44). Following this observation, it can be postulated that DTPA does not hinder the activity of chalcone moiety while maintaining chelation propensity. Furthermore, the DTPA chelator has a higher affinity for binding ^99m^Tc. This is evident from the dissociation constant values, wherein *K*_*d*_ for the ^99m^Tc complex is slightly higher than the ^68^Ga complex (3.46 ± 0.41 nM) owing to stronger chelation of ^99m^Tc to DTPA moiety. The data suggest a similar binding propensity, and therefore, the development of a more stable probe utilizing ^99m^Tc as the radiometal for chelation with DT(Ch)_2_.

For the blocking assay, DT(Ch)_2_ (5) and DMIC were used as the competing ligands. The results display Aβ_42_ aggregates binding reduction by ∼80% for ^99m^Tc–DT(Ch)_2_ when 100-fold excess of DT(Ch)_2_ (5) and DMIC are present. These results can be attributed to the high-binding affinity of the complex to the specific binding sites on the Aβ plaques. Studies indicate tolerability to structural modifications on the chalcone moiety, i.e., retainment of binding properties and binding sites of parent molecule following chelation. The results were comparable to a series of novel chalcone-mimic ^99m^Tc–cyclopentadienyl tricarbonyl complexes developed by Li et al. (17) with blocking assay values of 80%. Similar results were also observed for ^99m^Tc-radiolabeled 2-arylimidazo[2,1-b]benzothiazole derivatives, also developed for amyloid imaging (20).

### Human Serum Stability Studies

*In vitro* stability of purified ^99m^Tc–DT(Ch)_2_ in fresh human serum extract was analyzed *via* ITLC-SG under physiological pH. ^99m^Tc–DT(Ch)_2_ exhibited low transcomplexation of ^99m^Tc (2.79%) for the assay and the complex stability was maintained beyond 24 h (Supplementary Figure 4). The high stability of the ^99m^Tc–DT(Ch)_2_ complex in human serum assay substantiates the applicability of the complex for *in vivo* imaging.

### Blood Kinetics Evaluation

Pharmacokinetics was analyzed in normal rabbits by blood kinetics studies, where ^99m^Tc–DT(Ch)_2_ exhibits low-blood retention with 25.6% activity at 15 min and 8.65% at 2 h. Blood kinetics demonstrate a fast clearance rate before 1 h which decreased at the later time periods (Supplementary Figure 5) following a biphasic pattern with *t*_1/2_(*F*) = 30 min ± 0.09 and *t*_1/2_(*S*) = 4 h 20 min ± 0.06. Rapid clearance of the complex from the blood pool minimizes the background activity. A rapid complex clearance is also indicative of the absence of any non-specific binding or prolonged retention.

### Cytotoxicity Assay

The low cytotoxicity of chalcone derivatives toward normal cells is well-documented (45). Similar results were obtained for DT(Ch)_2_ ligand, wherein <17% toxicity was observed through MTT assay even after 48 h of incubation with HEK 293 cells. The cell toxicity increases with concentration, however, the increase is low and insignificant. Cell viability remains in the range of 88–95% up to 24 h, which ensures most of the ligand is flushed out of the system. Additionally, cell viability of around 85% is observed throughout the concentration range and observation time. The results of cytotoxicity are therefore encouraging and combined with the hemolysis profile provide a complete picture of high biocompatibility of the DT(Ch)_2_ ligand (Supplementary Figure 6).

### Hemolysis Assay

Red blood cells are the primary blood components interacting with the ligand. Any adverse effect of such direct interaction, such as rupturing of RBCs (hemolysis), is a toxic event and can limit the usage of that ligand for pharmaceutical purposes. The percentage of the released hemoglobin following cell lysis, therefore, is an indispensable standard test utilized in the assessment of viable hemocompatibility. The hemolytic assay was performed with PBS as negative control (pH = 7) and Triton X-100 as 100% hemolysis causing positive control. Equal amounts of RBCs suspended in varying concentrations (0.125–2 mg/mL) of ligand displayed <5% hemolysis, which is an acceptable standard (Supplementary Figure 7). The washout of the ligand from the body occurs within 4 h of administration, and there was no observable toxicity to the RBCs in this time frame. The results thus demonstrate high hemocompatibility and suitability of the ligand for *in vivo* administration.

### *In vivo* Scintigraphy and Biodistribution Studies

**FIGURE 7:**
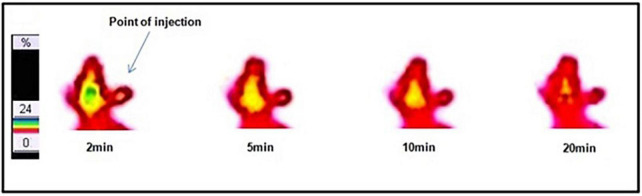
Dynamic lateral planar single photon emission computed tomography (SPECT) images of rabbit after i.v. injection of ^99m^Tc–DT(Ch)_2_.

The dynamic SPECT images in a normal rabbit brain following intravenous injection of ^99m^Tc–DT(Ch)_2_ (17.5 MBq) were obtained at the time interval of 2–20 min p.i. The images obtained were encouraging and showed a high-activity concentration in the brain at 2 min p.i., and as expected, the radiotracer showed a rapid washout (Figure 7). DT(Ch)_2_ demonstrated desirable pharmacokinetics, which is crucial for a higher signal-to-noise ratio. The results were quantified by biodistribution analysis wherein BALB/c mice were administered with ^99m^Tc–DT(Ch)_2_ (Supplementary Figure 8) and studied at 2–60 min post-injection. In the brain of healthy mice, the complex displayed a high initial concentration uptake followed by a fast washout, a property advantageous to the Aβ imaging agents. Consistent with the blood kinetics analysis in rabbits, the complex showed good plasma pharmacokinetics in mice as well as in rabbits where activity decreased rapidly from the blood. ^99m^Tc–DT(Ch)_2_ showed 1.16 ± 0.02% ID/g of activity in the brain at 2 min, which came down to 0.31 ± 0.01% ID/g at 30 min p.i. This indicates that the probe not only rapidly crosses BBB in normal mice but is also followed by a fast washout, as evident from the brain_2min_/brain_30min_ ratio of 3.74. The 2-min post-injection values are similar to that of the previously reported ^99m^Tc-based Aβ_1–42_-specific tracers, ^99m^Tc-BAT-chalcone (1.48% ID/g), ^99m^Tc-BAT-FL (0.64% ID/g), ^99m^Tc-BAT-AR (0.74% ID/g), series of [CpM(CO)_3_] derivatives (M = Re, ^99m^Tc) (0.50–0.26% ID/g), and 2-(4′-aminophenyl)benzothiazole derivative, ^99m^Tc-**1** (0.53% ID/g) (43, 46–48). The brain_2min_/brain_30min_ ratio for ^99m^Tc–DT(Ch)_2_ was also higher than a series of ^99m^Tc-labeled 2-arylbenzothiazoles (1.15–3.40) (49) and comparable to ^125^I derivatized allyloxy chalcone derivative, ^125^ I-**2** (1.9) (50). The uptake is attributed to the presence of chalcone moieties as ^99m^Tc–DTPA is known for low brain uptake (0.04% after 30 min). However, brain uptake and brain_2min_/brain_30min_ ratio was lower than some known amyloid-specific complexes such as PiB (ratio of brain_2min_/brain_30min_ as 12) (51), radioiodinated chalcones ^125^I-**4**, ^125^I-**7**, ^125^I-**10**, ^125^I-**14**, and ^125^I-**16** (2.0–4.7% ID/g at 2 min) (42), ^125^I-**3** (2.34% ID/g) (52), and chalcone derivative ^125^I-**14** (2.56% ID/g) (53). Additionally, ^99m^Tc complexes displaying higher initial uptake have also been previously reported such as cyclopentadienyl tricarbonyl complex ^99m^Tc-**3** (4.10 ± 0.38% ID/g), 2-arylbenzothiazole derivative ^99m^Tc-**14b** (2.11% ID/g), and oligoethyleneoxy-modified ^99m^Tc-**15** (2.10% ID/g) (17, 49, 54). The lower uptake can be due to the hydrophilic nature of the DTPA chelator. This chalcone-derivatized structural alternative, therefore, has the potential for further fine-tuning necessary for a SPECT amyloid imaging agent.

The labeled complex showed the highest activity accumulation in the liver (10–11% ID/g) with a very slow washout, a pattern independent of the radioisotope used. Additionally, activity depletion in blood was also rapid indicating good plasma pharmacokinetics. The activity was excreted *via* kidneys and liver suggesting both hepatobiliary and renal modes of excretion. High lipophilicity of the tracer can explain high uptake in the liver and is also a hallmark of chalcone derivatives reported earlier. Importantly, the uptake in other non-targeted organs (such as the spleen, the lungs, and the heart) was minimal.

**FIGURE 8:**
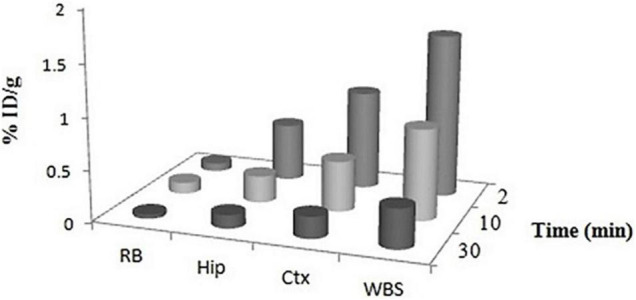
Regional uptake of ^99m^Tc–DT(Ch)_2_ in mice brain. WBS, whole brain section; Ctx, cortex; Hip, hippocampus; RB, rest of brain at 2, 10, and 30 min post-injection.

Regional brain distribution was also evaluated to study the percentage uptake of ^99m^Tc–DT(Ch)_2_ in different brain regions (Figure 8). The results suggested the highest activity accumulation of the complex in the cortex (∼40–45%) and hippocampus (∼25–30%) regions, which are crucial for the formation of new memory and are the most affected brain regions in Alzheimer’s disease (55).

Furthermore, to evaluate the *in vivo* amyloid-binding affinity, an amyloid-overexpressing rodent model was used. The formation of amyloid plaques was confirmed through Congo Red staining (wavelength = 614 nm) and Thioflavin S staining (wavelength = 450 nm) (Supplementary Figure 9) in accordance with previous literature (56). The amyloid-overexpressing model displayed 40% higher tracer uptake compared to the control model when checked through biodistribution studies. The results indicate enhanced binding in presence of the amyloid plaques, thus confirming the binding affinity of ^99m^Tc–DT(Ch)_2_.

## Conclusion

Memory decline and cognitive deficit are the most profound and disabling consequences of TBI and confer secondary risk of AD. The study here explores the bio-evaluation of ^99m^Tc-complexed DT(Ch)_2_ as a potential amyloid-targeted Aβ SPECT imaging agent. The complexation with ^99m^Tc was carried out in a facile single-step process with high purity and efficiency. The molecular modeling experiments aid in the understanding of the binding interactions of the DTPA-conjugated ligand with the amyloid protein displaying thermodynamically favorable interactions. The complex displayed a high-Aβ aggregates binding affinity during *in vitro* evaluation and a rapid washout from the brain in normal rabbits and mice. With lipophilicity in the range permitted to cross the BBB, the complex displayed a good initial uptake with high localization in the as-reported most affected area in the AD brain, cerebellum, and hippocampus region of the normal mice. The ligand offers a suitable framework for the development of a cost-effective and versatile amyloid diagnostic agent for the ^99m^Tc-based SPECT, thus increasing the availability of Aβ diagnosis at more hospitals.

## Data Availability Statement

The original contributions presented in this study are included in the article/Supplementary Material, further inquiries can be directed to the corresponding author.

## Ethics Statement

The studies involving human participants were reviewed and approved by Institutional Human Ethical Committee. The patients/participants provided their written informed consent to participate in this study. The animal study was reviewed and approved by Institutional Animal Ethics Committee under the guidelines of CPCSEA (Committee for the Purpose of Control and Supervision of Experiments on Animals), Government of India.

## Author Contributions

GM and KC: methodology, formal analysis, and writing the original draft. VK, SD, and NK: data analysis and methodology. MT: supervision and project administration. AD: conception, design, supervision, and funding acquisition. All authors have contributed to the article and approved the submitted version.

## Conflict of Interest

The authors declare that the research was conducted in the absence of any commercial or financial relationships that could be construed as a potential conflict of interest.

## Publisher’s Note

All claims expressed in this article are solely those of the authors and do not necessarily represent those of their affiliated organizations, or those of the publisher, the editors and the reviewers. Any product that may be evaluated in this article, or claim that may be made by its manufacturer, is not guaranteed or endorsed by the publisher.
