# Supplementary material for: Bio-Evaluation of 99mTc-Labeled Homodimeric Chalcone Derivative as Amyloid-β-Targeting Probe
Source: Front Med (Lausanne). 2022 Jun 17;9:813465. doi: 10.3389/fmed.2022.813465 (PMC9249127; doi:10.3389/fmed.2022.813465)
Supplement: Supplementary file 1 [file Data_Sheet_1.pdf]

Supplementary Material

- **Figure S1**. Synthetic scheme of $DT(Ch)_2$
- **Figure S2. (a)** ^{99m}Tc complexation of $DT(Ch)_2$; **(b)** HPLC chromatogram of ^{99m}Tc-$DT(Ch)_2$
- **Figure S3**. In vitro serum stability of ^{99m}Tc-$DT(Ch)_2$ at 37°C (n=3)
- **Figure S4.** Saturation curve of ligand $DT(Ch)_2$
- **Figure S5.** Blood kinetics of ^{99m}Tc-$DT(Ch)_2$ administered through ear vein in normal rabbit
- **Figure S6.** Cytotoxicity assay of ligand $DT(Ch)_2$
- **Figure S7.** Haemolysis assay of ligand $DT(Ch)_2$
- **Figure S8.** Biodistribution of ^{99m}Tc-$DT(Ch)_2$ following tracer administration in normal and amyloid overexpressing mice (n = 3) (Represented as % injected dose per gram)
- **Figure S9.** Staining of amyloid brain sections with amyloid specific dye **(a)** Congo Red **(b)** Thioflavin S
- **Characterization Data**
- **Figure S10.** ^{1}H NMR spectrum of tert-Butyl 2-bromoethylcarbamate (1)
- **Figure S11.** ^{13}C NMR spectrum of tert-Butyl 2-bromoethylcarbamate (1)
- **Figure S12.** ESI-MS spectrum of tert-Butyl 2-bromoethylcarbamate (1)
- **Figure S13.** ^{1}H NMR spectrum of (E)-1-(4-hydroxyphenyl)-3-(4-isopropylphenyl)prop-2-en-1-one (2)
- **Figure S14.** ^{13}C NMR spectrum of (E)-1-(4-hydroxyphenyl)-3-(4-isopropylphenyl)prop-2-en-1-one (2)
- **Figure S15.** ESI-MS spectrum of (E)-1-(4-hydroxyphenyl)-3-(4-isopropylphenyl)prop-2-en-1-one (2)

10.3389/fmed.2022.813465

- **Figure S16.** ^{1}H NMR spectrum of (E)-*tert*-butyl 2-(4-(3-(4-(dimethylamino)phenyl)acryloyl)phenoxy)ethylcarbamate (3)
- **Figure S17.** ^{13}C NMR spectrum of (E)-*tert*-butyl 2-(4-(3-(4-(dimethylamino)phenyl)acryloyl)phenoxy)ethylcarbamate (3)
- **Figure S18.** ESI-MS spectrum of (E)-*tert*-butyl 2-(4-(3-(4-(dimethylamino)phenyl)acryloyl)phenoxy)ethylcarbamate (3)
- **Figure S19.** ^{1}H NMR spectrum of (E)-1-(4-(2-aminoethoxy)phenyl)-3-(4-(dimethylamino)phenyl)prop-2-en-1-one (4)
- **Figure S20**. ^{13}C NMR spectrum of (E)-1-(4-(2-aminoethoxy)phenyl)-3-(4-(dimethylamino)phenyl)prop-2-en-1-one (4)
- **Figure S21.** ESI-MS spectrum of (E)-1-(4-(2-aminoethoxy)phenyl)-3-(4-(dimethylamino)phenyl)prop-2-en-1-one (4)
- **Figure S22.** ^{1}H NMR spectrum of 6,9-bis(carboxymethyl)-14-(4-((E)-3-(4-(dimethylamino)phenyl)acryloyl)phenoxy)-3-(2-((2-(4-((E)-3-(4-(dimethylamino)phenyl)acryloyl)phenoxy)ethyl)amino)-2-oxoethyl)-11-oxo-3,6,9,12-tetraazatetradecanoic acid (5)
- **Figure S23.** ^{13}C NMR spectrum of 6,9-bis(carboxymethyl)-14-(4-((E)-3-(4-(dimethylamino)phenyl)acryloyl)phenoxy)-3-(2-((2-(4-((E)-3-(4-(dimethylamino)phenyl)acryloyl)phenoxy)ethyl)amino)-2-oxoethyl)-11-oxo-3,6,9,12-tetraazatetradecanoic acid (5)
- **Figure S24.** ESI-MS spectrum of 6,9-bis(carboxymethyl)-14-(4-((E)-3-(4-(dimethylamino)phenyl)acryloyl)phenoxy)-3-(2-((2-(4-((E)-3-(4-(dimethylamino)phenyl)acryloyl)phenoxy)ethyl)amino)-2-oxoethyl)-11-oxo-3,6,9,12-tetraazatetradecanoic acid (5)

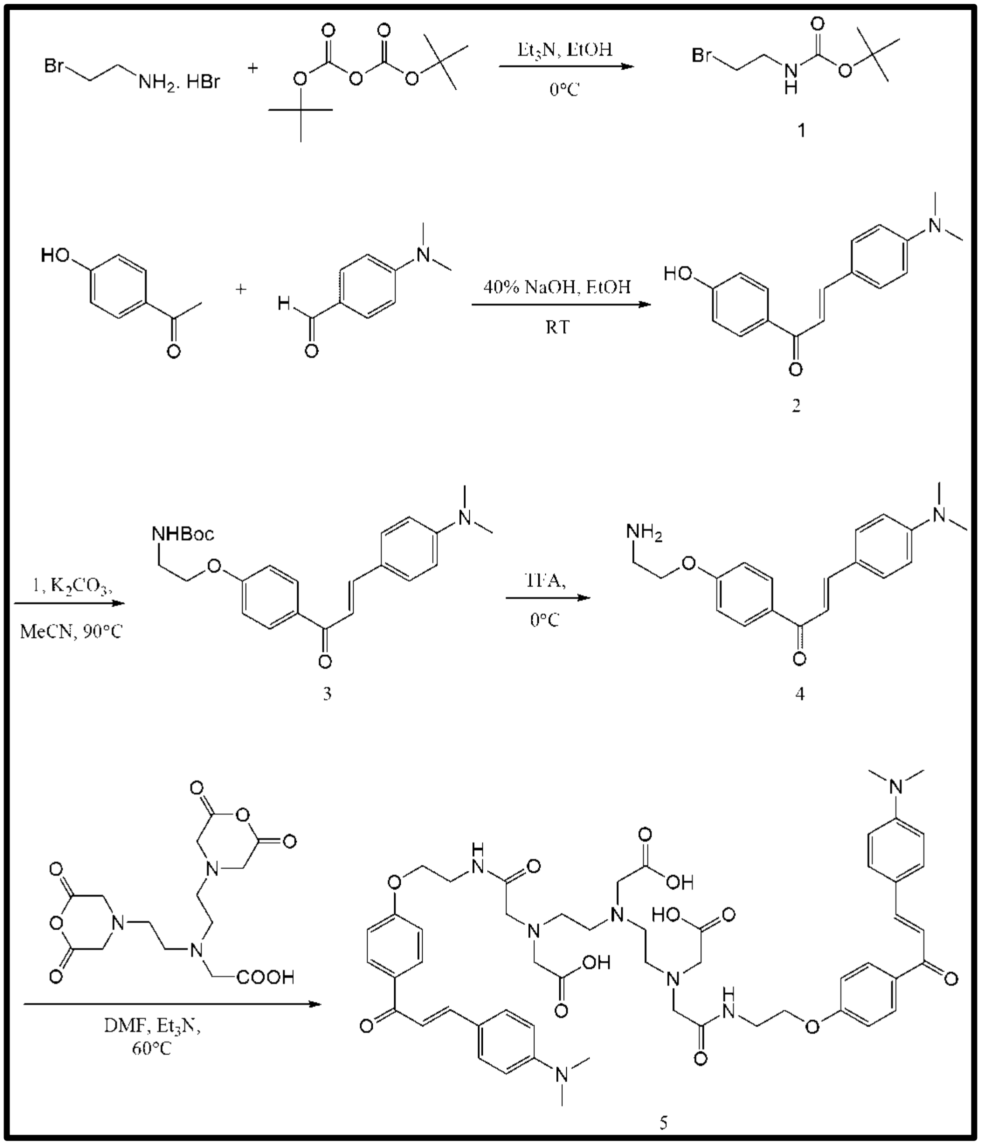

Figure S1. Synthetic scheme of $DT(Ch)_2$

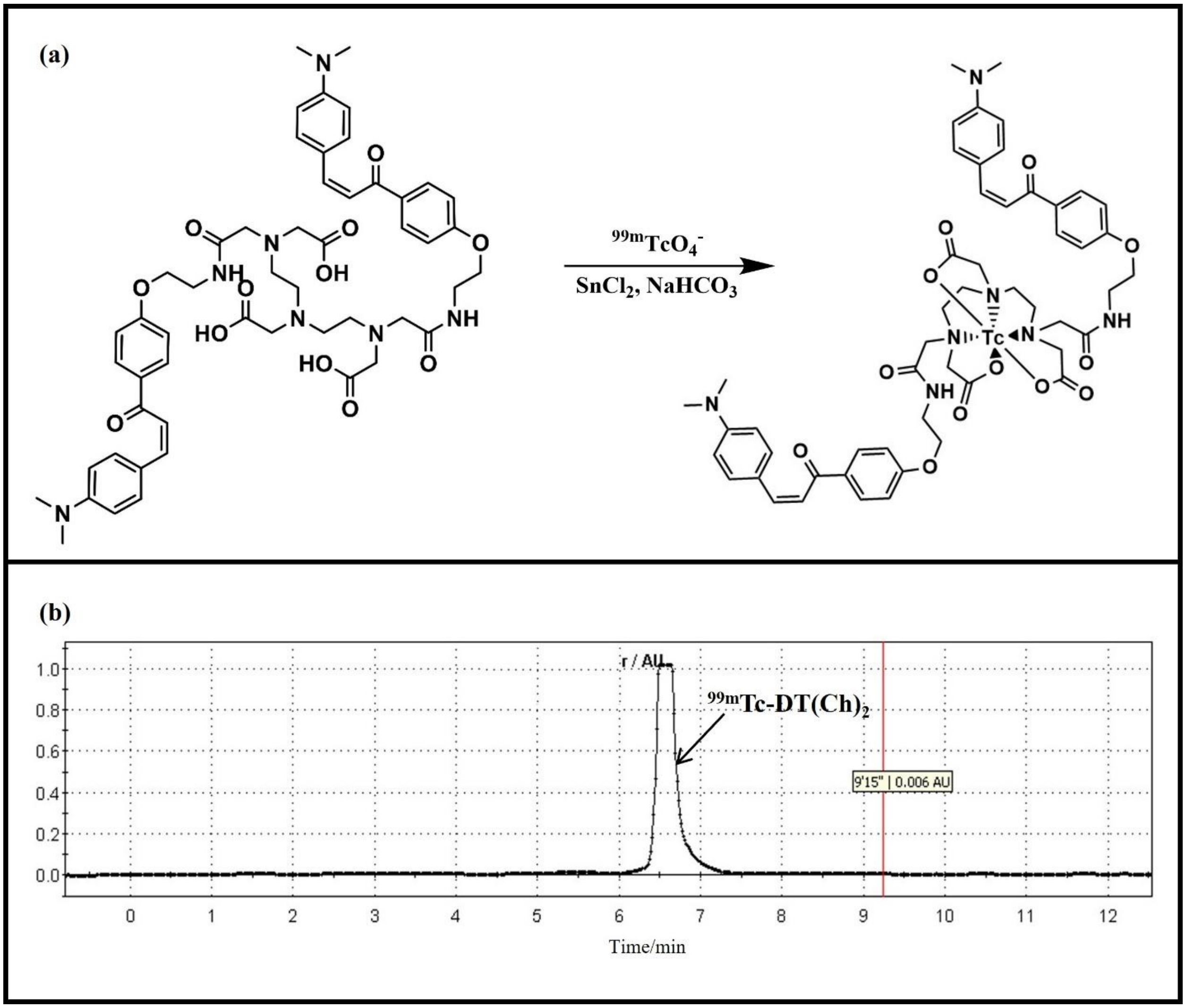

Figure S2. (a) ^{99m}Tc complexation of DT(Ch)$_2$; **(b)** HPLC chromatogram of ^{99m}Tc-DT(Ch)$_2$.

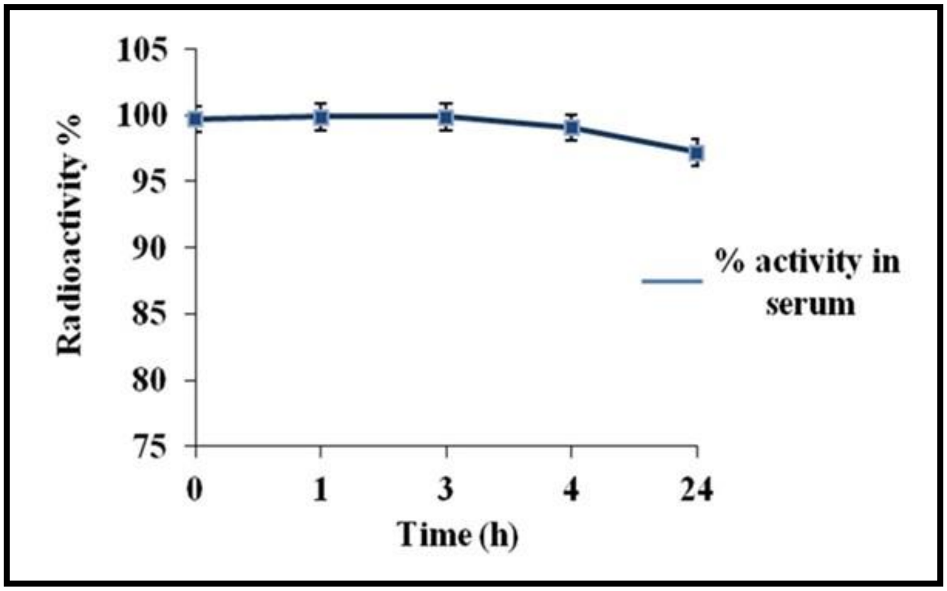

Figure S3. In vitro serum stability of ^{99m}Tc-DT(Ch)$_2$ at 37°C (n=3)

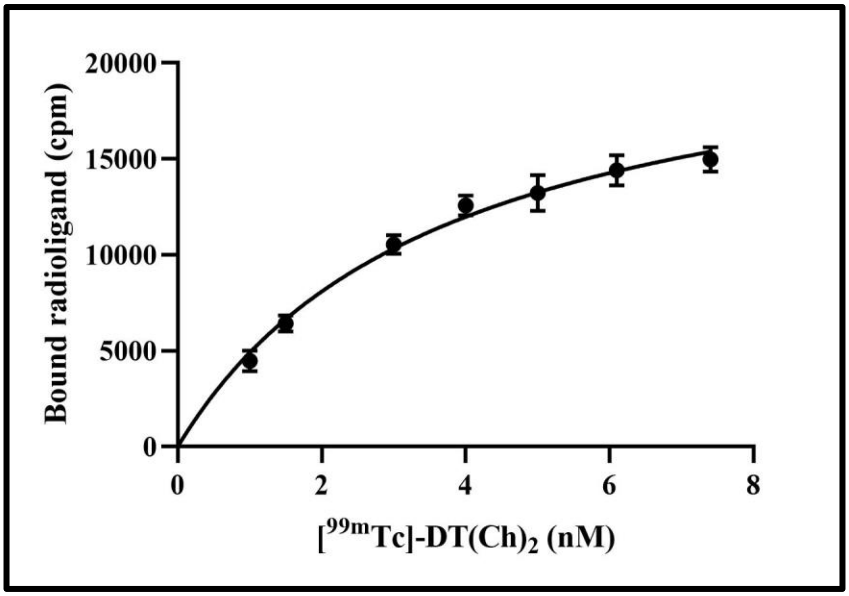

Figure S4. Saturation curve of ligand $DT(Ch)_2$

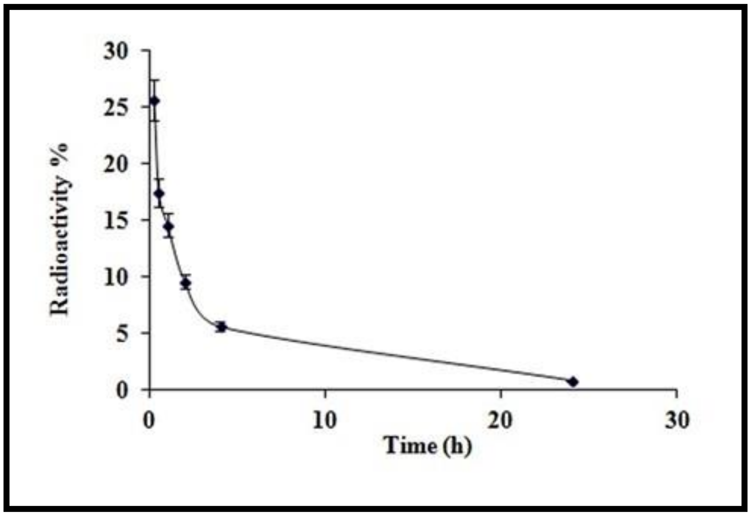

Figure S5. Blood kinetics of ^{99m}Tc-DT(Ch)$_2$ administered through ear vein in normal rabbit

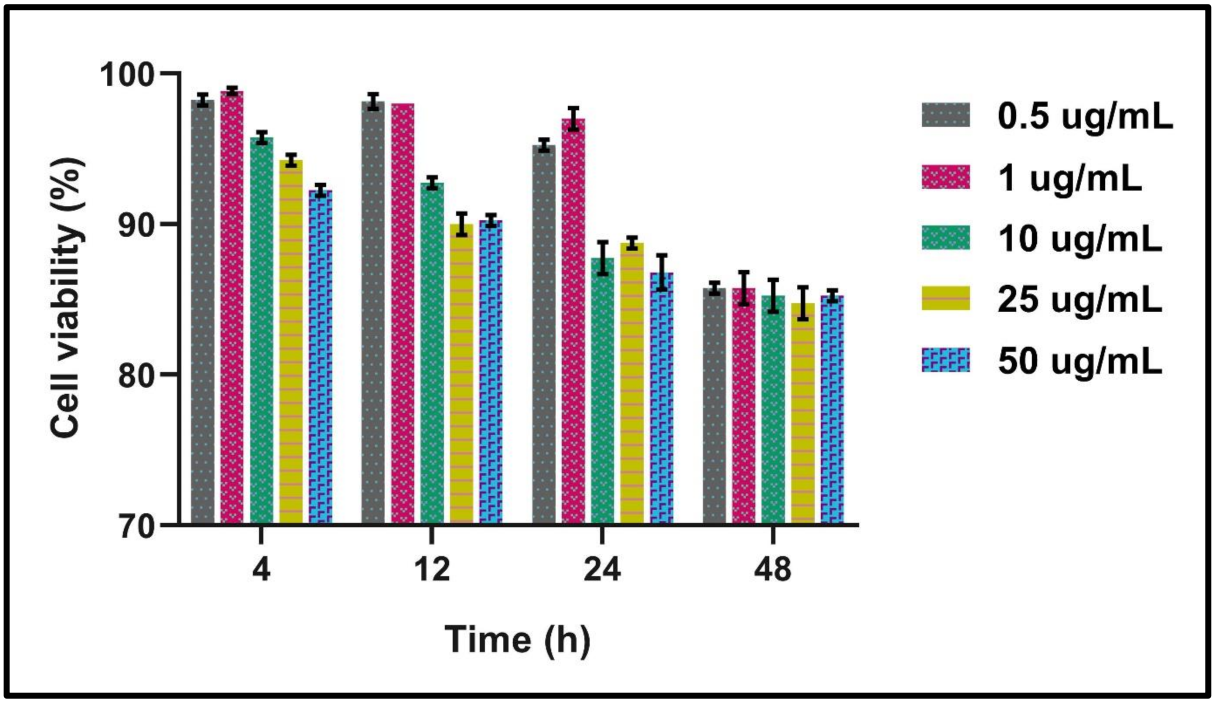

Figure S6. Cytotoxicity assay of ligand $DT(Ch)_2$

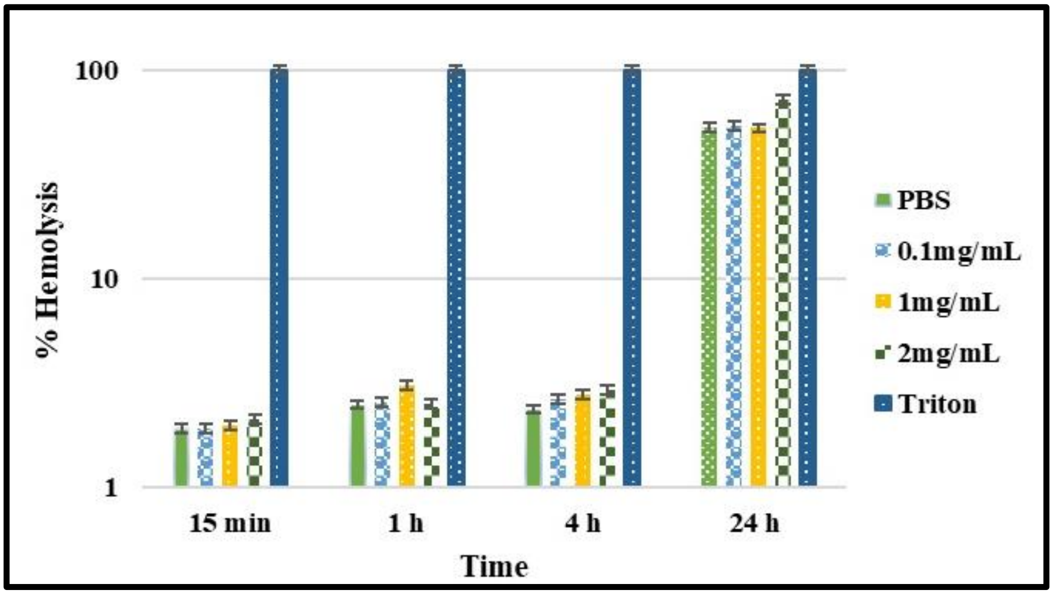

Figure S7. Hemolysis assay of ligand $DT(Ch)_2$

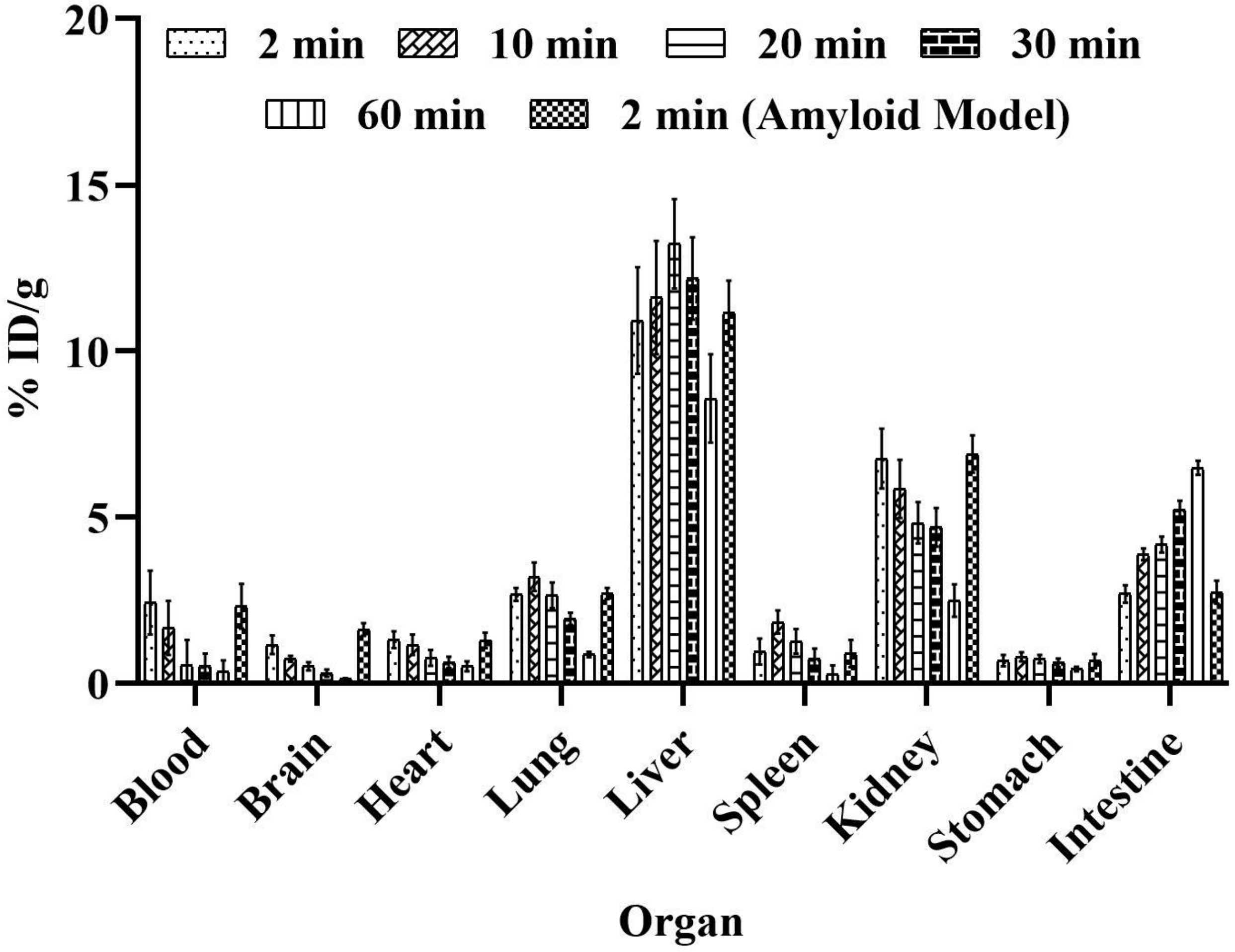

Figure S8. Biodistribution of ^{99m}Tc-DT(Ch)$_2$ following tracer administration in normal and amyloid overexpressing mice (n = 3) (Represented as % injected dose per gram)

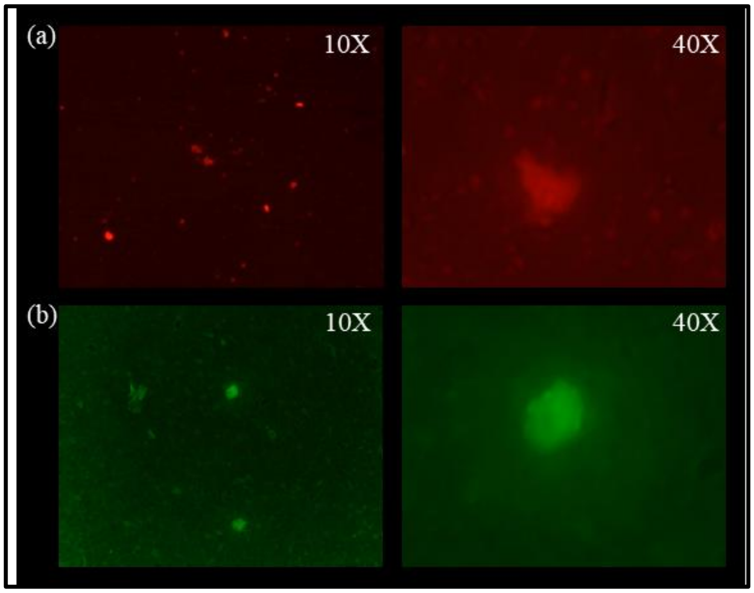

Figure S9. Staining of amyloid brain sections with amyloid specific dye **(a)** Congo Red **(b)** Thioflavin S

Characterization Data:

The synthesis of the ligand was carried out according to the previously reported procedure (1).

i. *tert*-Butyl 2-bromoethylcarbamate (1)

^{1}H NMR (400 MHz, $CDCl_3$): δ 1.30 (s, 9H), 3.33 (t, J=5.6 Hz, 2H), 3.52 (t, J=5.6, 2H), 5.22 (s, 1H). ^{13}C NMR (100 MHz, $CDCl_3$): δ 28.33, 41.48, 42.96, 79.82, 155.59. Elemental analysis calcd for $C_7H_{14}BrNO_2$: C, 37.52; H, 6.30; N, 6.25. Found: C, 37.24; H, 6.28; N, 6.21. MS(ESI): m/z calcd $[M + H]^+$: 224, found: 224 $[M + H]^+$

ii. (E)-1-(4-Hydroxyphenyl)-3-(4-isopropylphenyl)prop-2-en-1-one (2)

^{1}H NMR (400 MHz, $CDCl_3$):δ 3.01 (s, 6H), 6.68 (d, J=8.8 Hz, 2H), 6.88 (d, J=8.8 Hz, 2H), 7.31 (d, J=15.2 Hz, 1H), 7.53 (d, J=9.2 Hz, 2H), 7.75 (d, J=15.2 Hz, 1H), 7.97 (d, J=8.8 Hz, 2H). ^{13}C NMR (100 MHz, CD_3OD): δ 38.85, 111.63, 114.91, 115.65, 122.60, 130.11, 130.20, 130.65, 145.36, 152.44, 162.13, 189.71. Elemental analysis calcd for $C_{17}H_{17}NO_2$: C, 76.38; H, 6.41; N, 5.24. Found: C, 75.52; H, 6.39; N, 5.20. MS(ESI): *m/z* calculated $[M+ H]^+$: 268, found 268 $[M+ H]^+$

iii. (E)-*tert*-Butyl-2-(4-(3-(4-(dimethylamino)phenyl)acryloyl)phenoxy)ethylcarbamate (3)

^{1}H NMR (400 MHz, $CDCl_3$): δ 1.49 (s, 9H), 3.04 (s, 6H), 3.59 (t, J=5.2 Hz, 2H), 4.13 (t, d=5.2 Hz, 2H), 6.72 (d, J=8.8, 2H), 6.98 (d, J=8.8 Hz, 2H), 7.34 (d, J=15.2, 1H), 7.55 (d, J=8.8, 2H), 7.81 (d, J=15.6, 1H), 8.03 (d, J=8.8, 2H). ^{13}C NMR (100 MHz, CD_3OD): δ 27.33, 38.84, 39.47, 66.82, 78.87, 111.62, 114.05, 115.54, 122.52, 130.29, 130.39, 131.44, 145.73, 152.50, 162.75, 189.68. Elemental

analysis calcd for $C_{24}H_{30}N_2O_4$: C, 70.22; H, 7.37; N, 6.82. Found: C, 69.4; H, 7.25; N, 6.56. MS(ESI): *m/z* calcd $[M + H]^+$: 411, found 411 $[M + H]^+$

iv. (E)-1-(4-(2-Aminoethoxy)phenyl)-3-(4-(dimethylamino)phenyl)prop-2-en-1-one (4)

^{1}H-NMR (400 MHz, D_2O) δ 7.85 (d, J = 8.1 Hz, 2H), 7.48-7.56 (m, 3H), 7.25 (d, J = 16.2 Hz, 1H), 6.97 (d, J = 8.4 Hz, 2H), 6.73 (d, J = 7.8 Hz, 2H), 4.22 (t, J = 4.7 Hz, 2H), 3.32 (t, J = 4.9 Hz, 2H), 2.83 (s, 6H). ^{13}C NMR (100 MHz, CD_3COCD_3): δ 38.74, 111.68, 115.05, 115.74, 122.23, 130.17, 130.39, 131.05, 145.33, 152.61, 162.72, 189.38. Elemental analysis calcd for $C_{19}H_{22}N_2O_2$: C, 73.52; H, 7.14; N, 9.03. Found: C, 72.84; H, 7.02; N, 8.80. MS(ESI): *m/z* calcd $[M+ H]^+$: 311, found 311 $[M+ H]^+$.

v. 6,9-bis(carboxymethyl)-14-(4-((E)-3-(4-(dimethylamino)phenyl)acryloyl)phenoxy)-3-(2-((2-(4-((E)-3-(4-(dimethylamino)phenyl)acryloyl)phenoxy)ethyl)amino)-2-oxoethyl)-11-oxo-3,6,9,12-tetraazatetradecanoic acid ($DT(Ch)_2$) (5)

^{1}H NMR (400 MHz, D_2O): δ 2.58-3.26 (m, 28H), 3.46 (br, 4H), 3.89 (br, 4H), 6.25 (d, J = 6Hz, 4H), 6.73-6.85 (m, 6H), 7.07 (d, J = 9 Hz, 4H), 7.27 (d, J = 15 Hz, 2H), 7.59 (d, J = 9 Hz, 4H). ^{13}C NMR (100 MHz, DMSO-d_6): δ 38.31, 40.24, 52.21, 55.31, 57.58, 58.15, 66.76, 112.20, 114.80, 116.46, 122.57, 130.99, 131.84, 144.80, 152.32, 162.34, 171.27, 173.16, 187.44. Elemental analysis calcd for $C_{52}H_{63}N_7O_{12}$: C, 63.85; H, 6.49; N, 10.02. Found: C, 63.60; H, 6.32; N, 9.75. MS(ESI): m/z calcd $[M + 2H]^+$: 979, found 979 $[M + 2H]^+$.

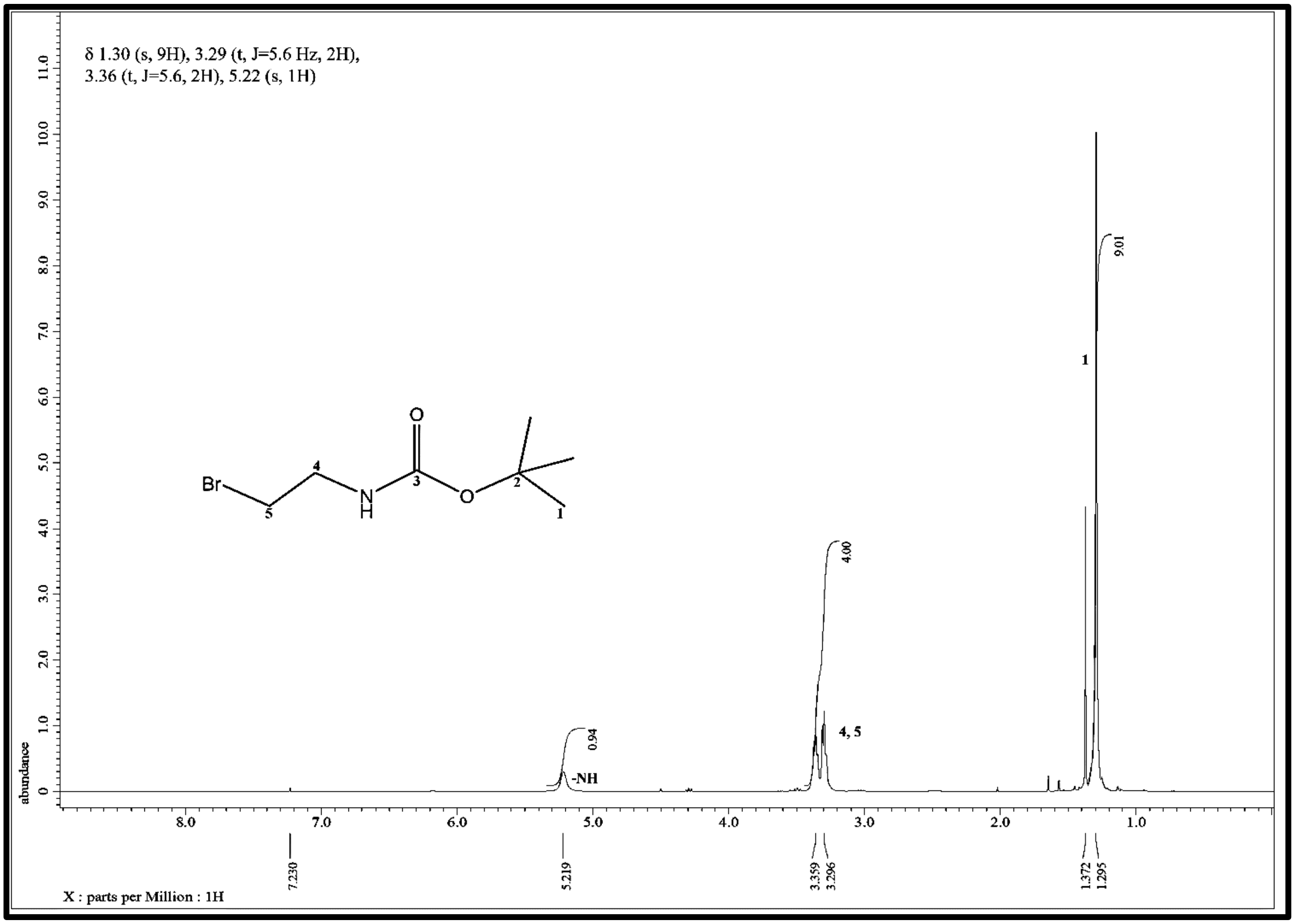

Figure S10. ^{1}H NMR spectrum of tert-Butyl 2-bromoethylcarbamate (1)

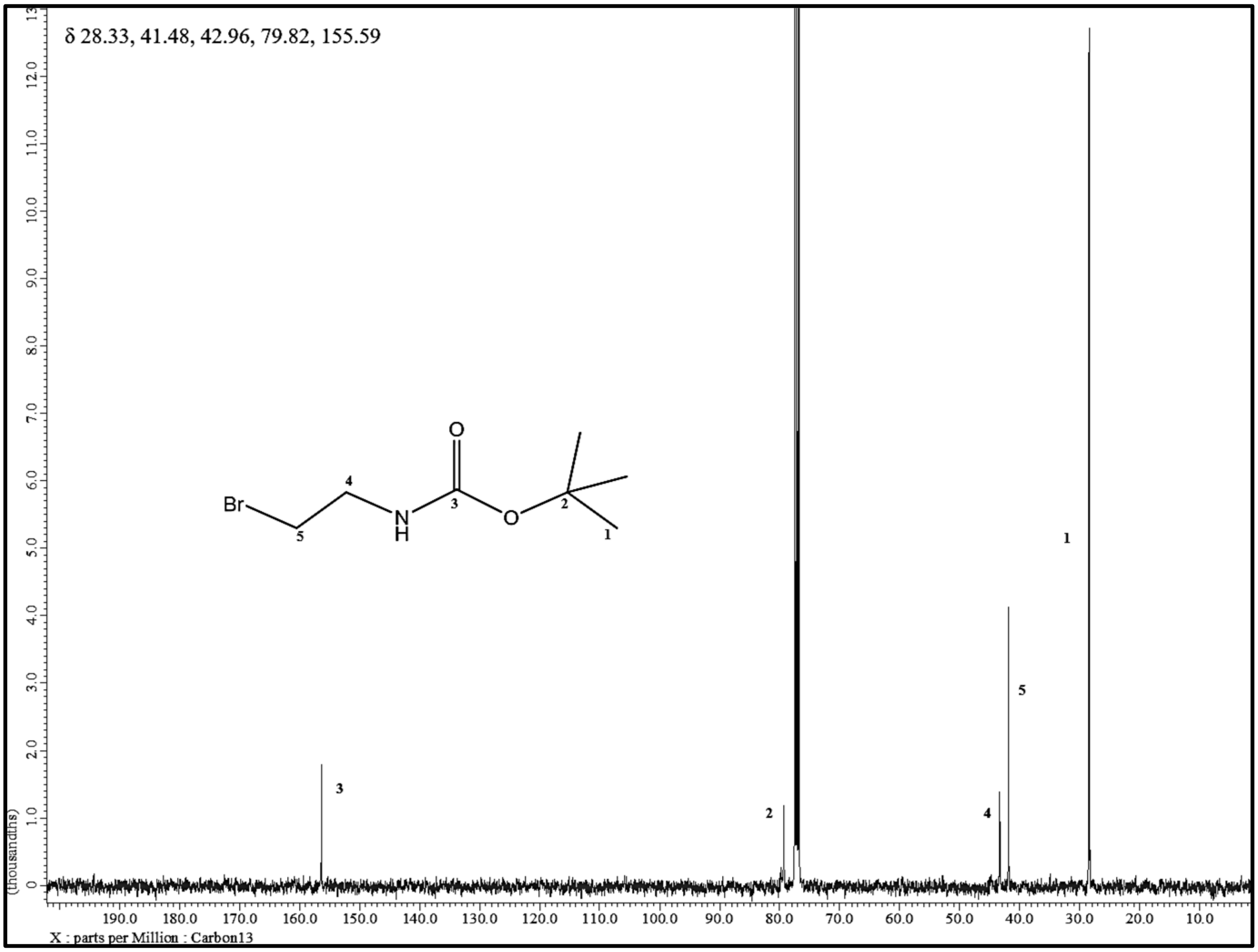

Figure S11. ^{13}C NMR spectrum of tert-Butyl 2-bromoethylcarbamate (1)

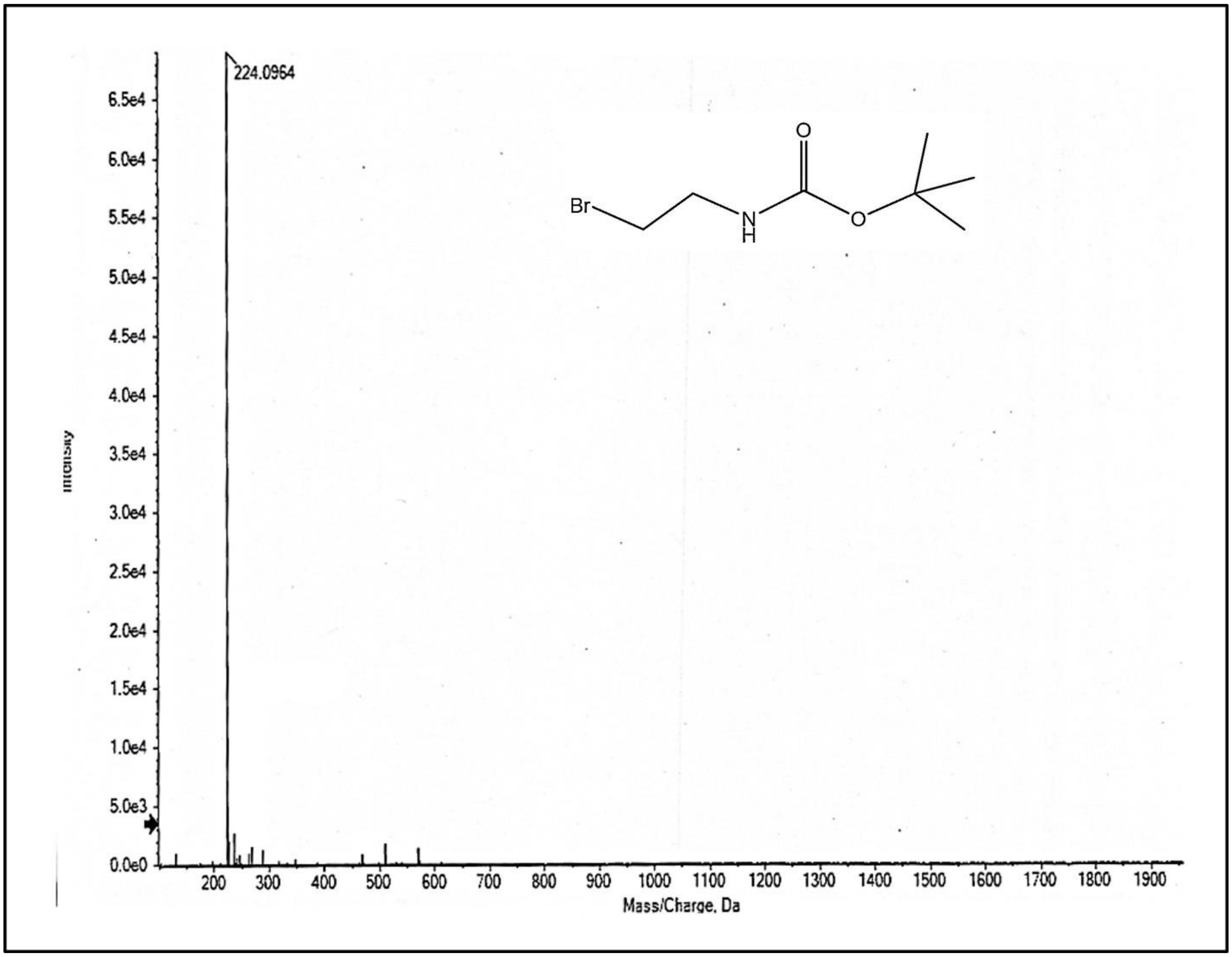

Figure S12. ESI-MS spectrum of tert-Butyl 2-bromoethylcarbamate (1)

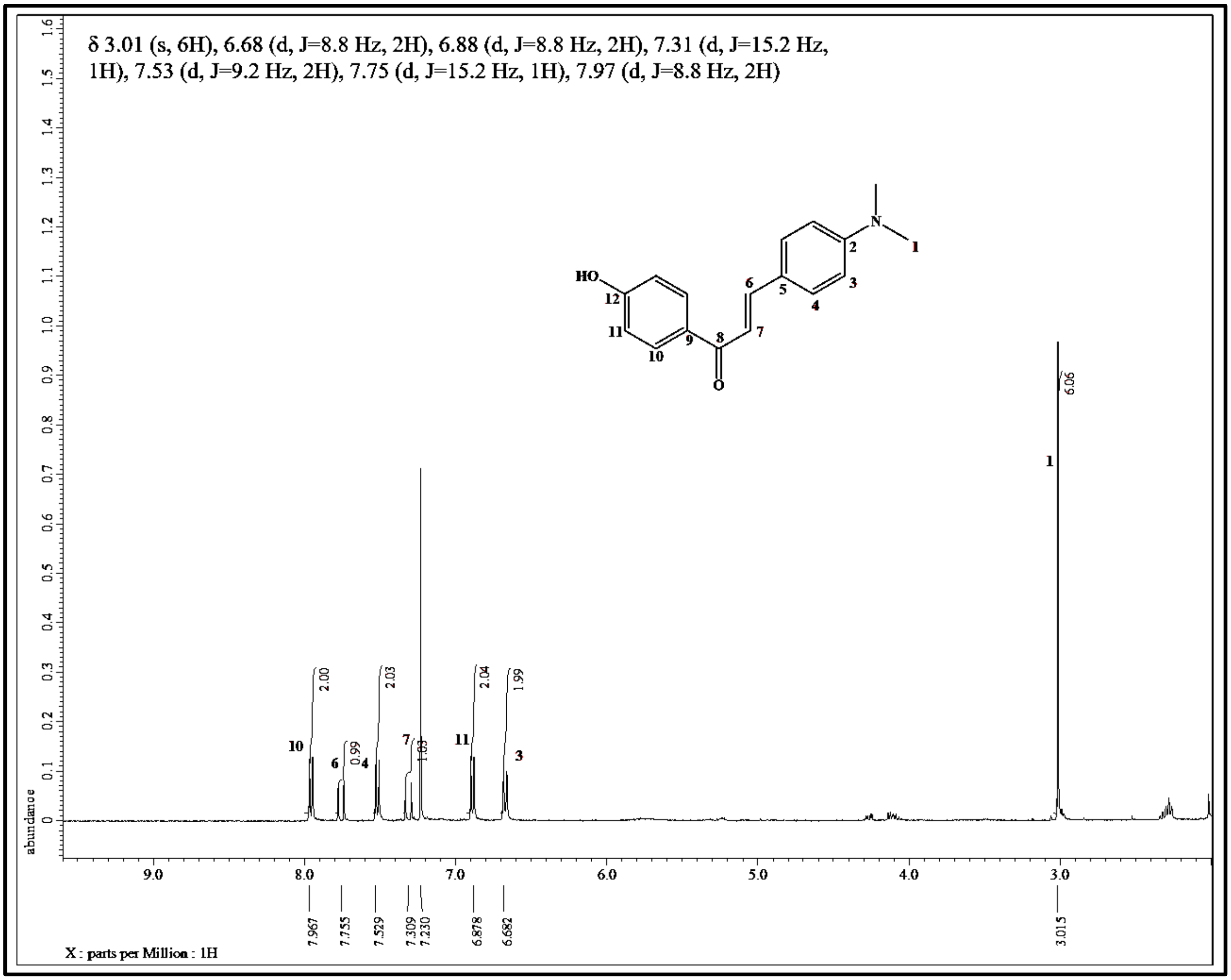

Figure S13. ^{1}H NMR spectrum of (E)-1-(4-hydroxyphenyl)-3-(4-isopropylphenyl)prop-2-en-1-one (2)

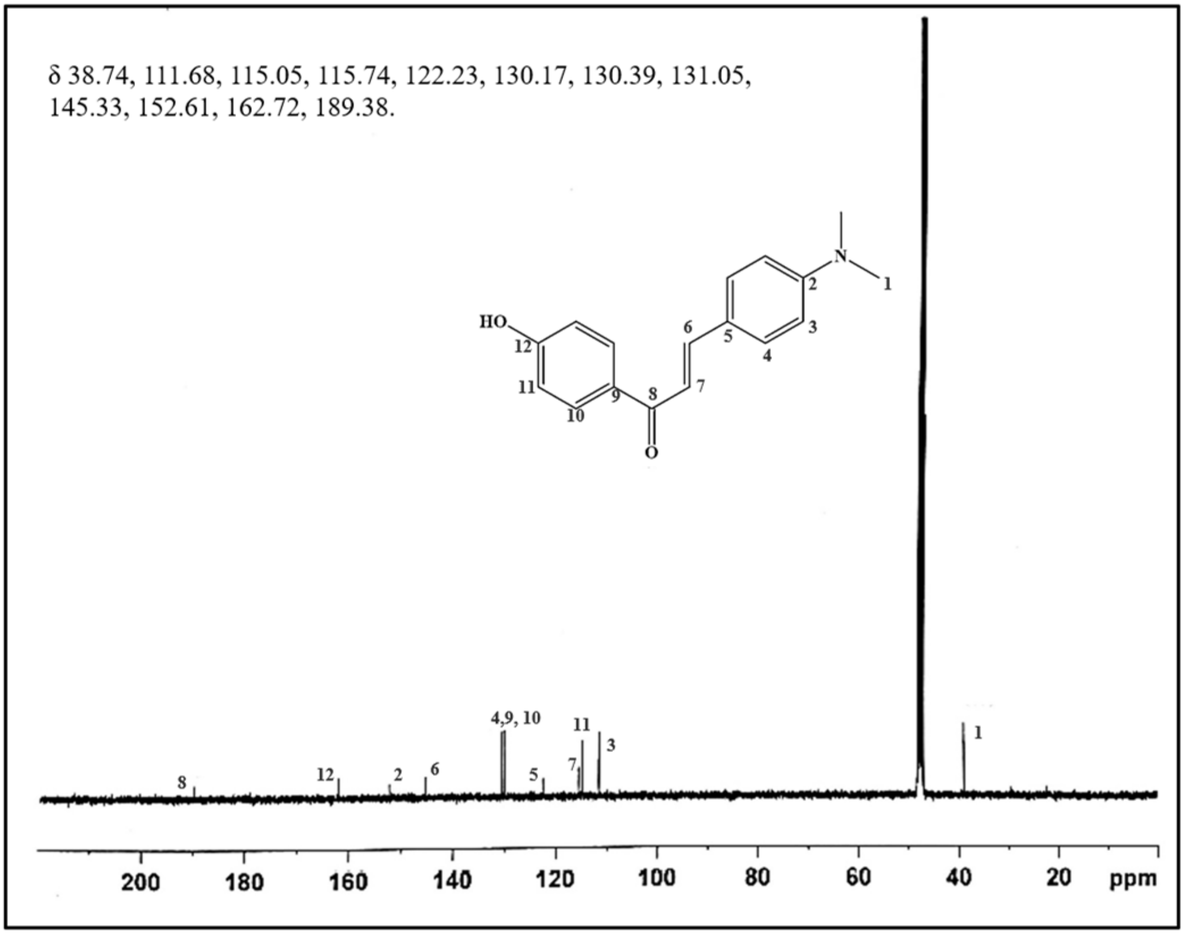

Figure S14. ^{13}C NMR spectrum of (E)-1-(4-hydroxyphenyl)-3-(4-isopropylphenyl)prop-2-en-1-one (2)

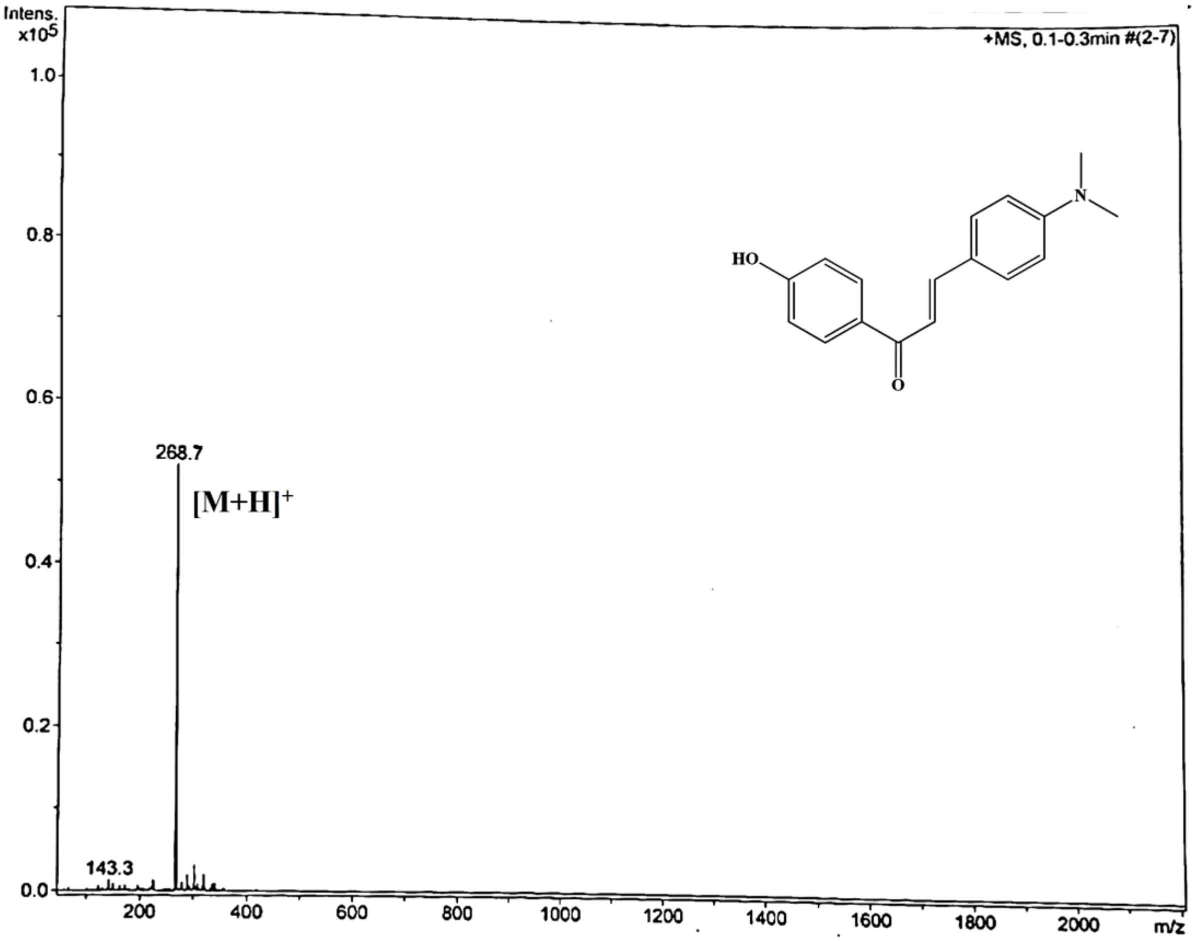

Figure S15. ESI-MS spectrum of (E)-1-(4-hydroxyphenyl)-3-(4-isopropylphenyl)prop-2-en-1-one (2)

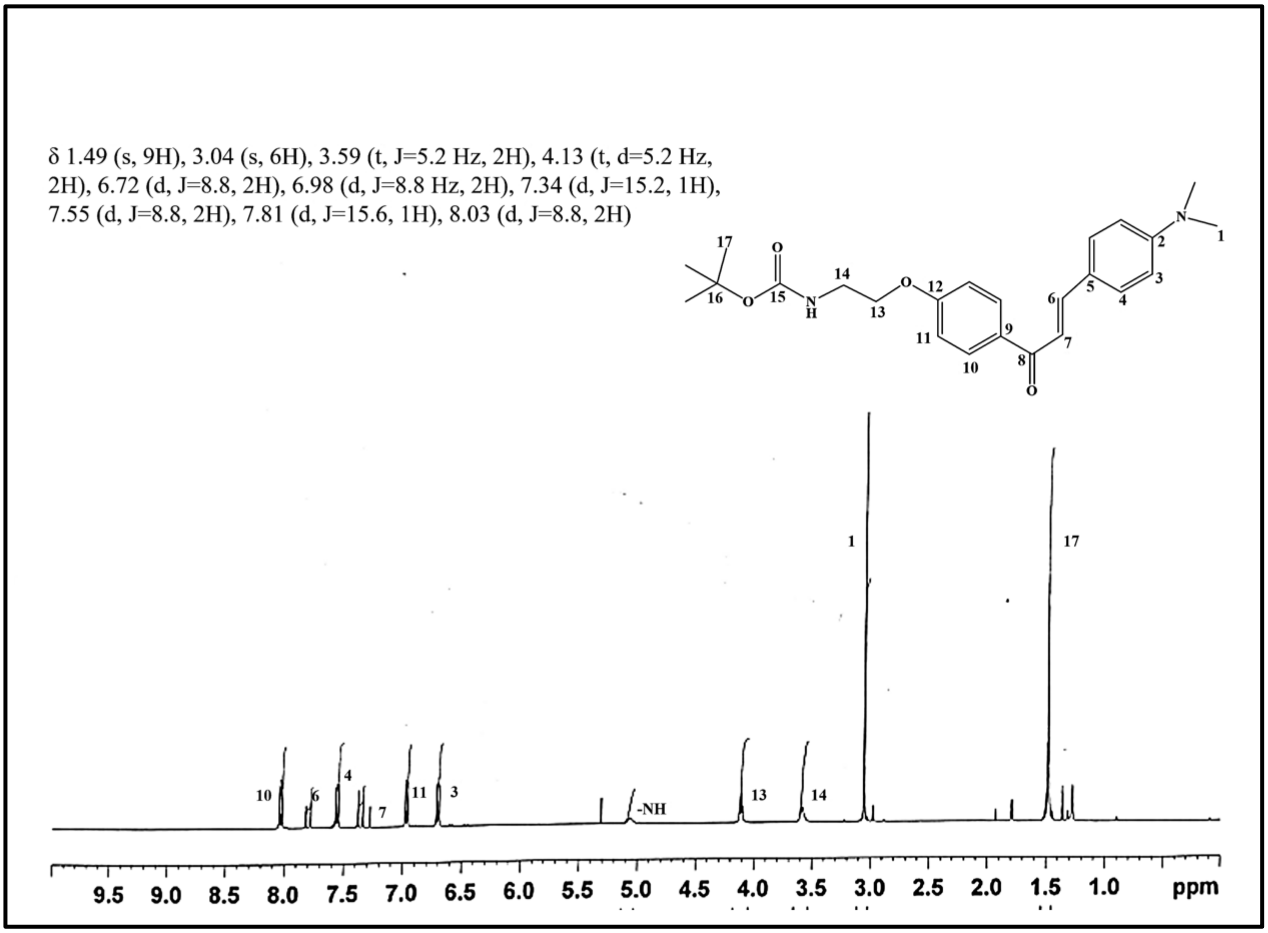

Figure S16. ^{1}H NMR spectrum of (E)-*tert*-butyl 2-(4-(3-(4-(dimethylamino)phenyl)acryloyl)phenoxy)ethylcarbamate (3)

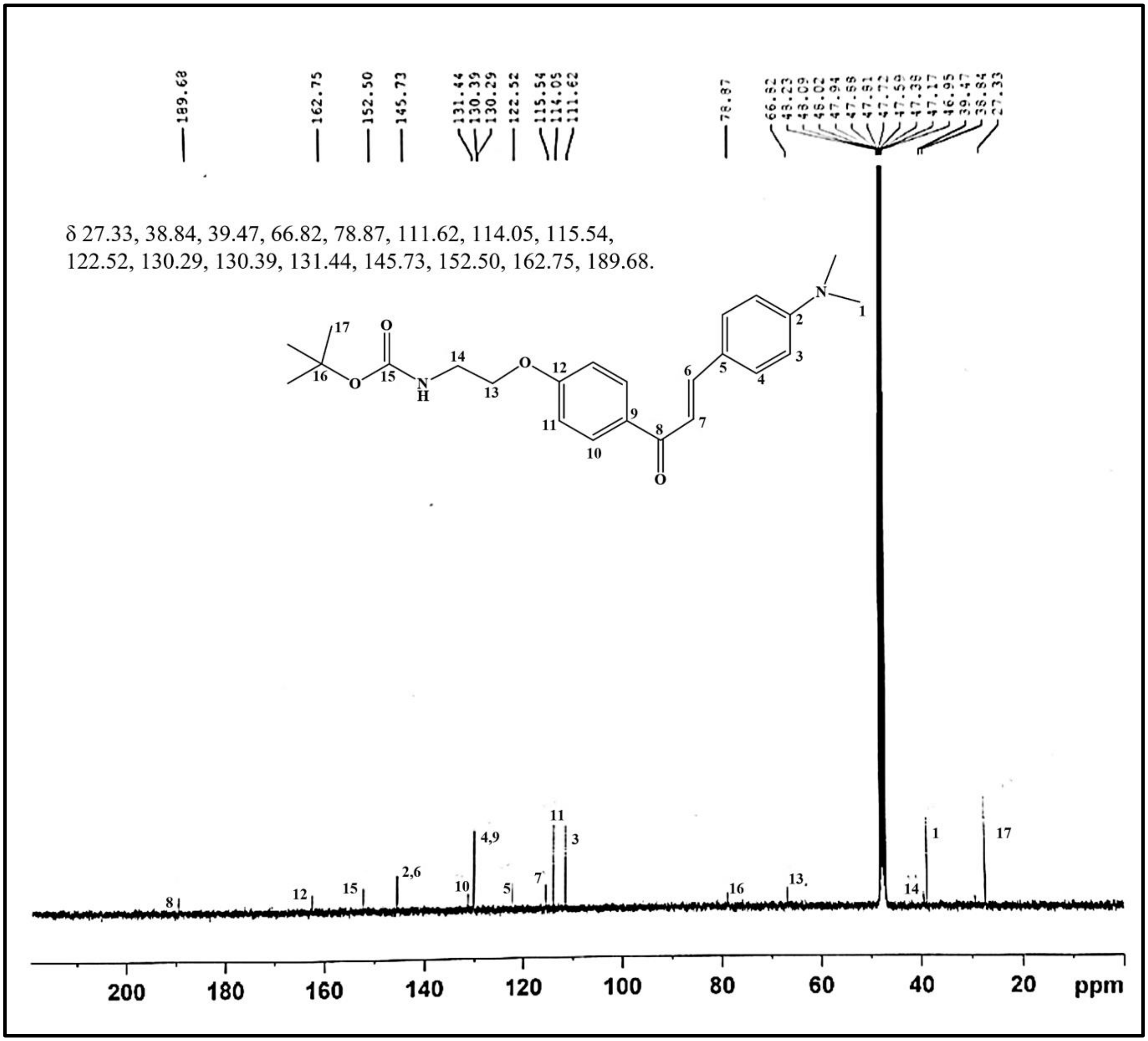

Figure S17. ^{13}C NMR spectrum of (E)-*tert*-butyl 2-(4-(3-(4-(dimethylamino)phenyl)acryloyl)phenoxy)ethylcarbamate (3)

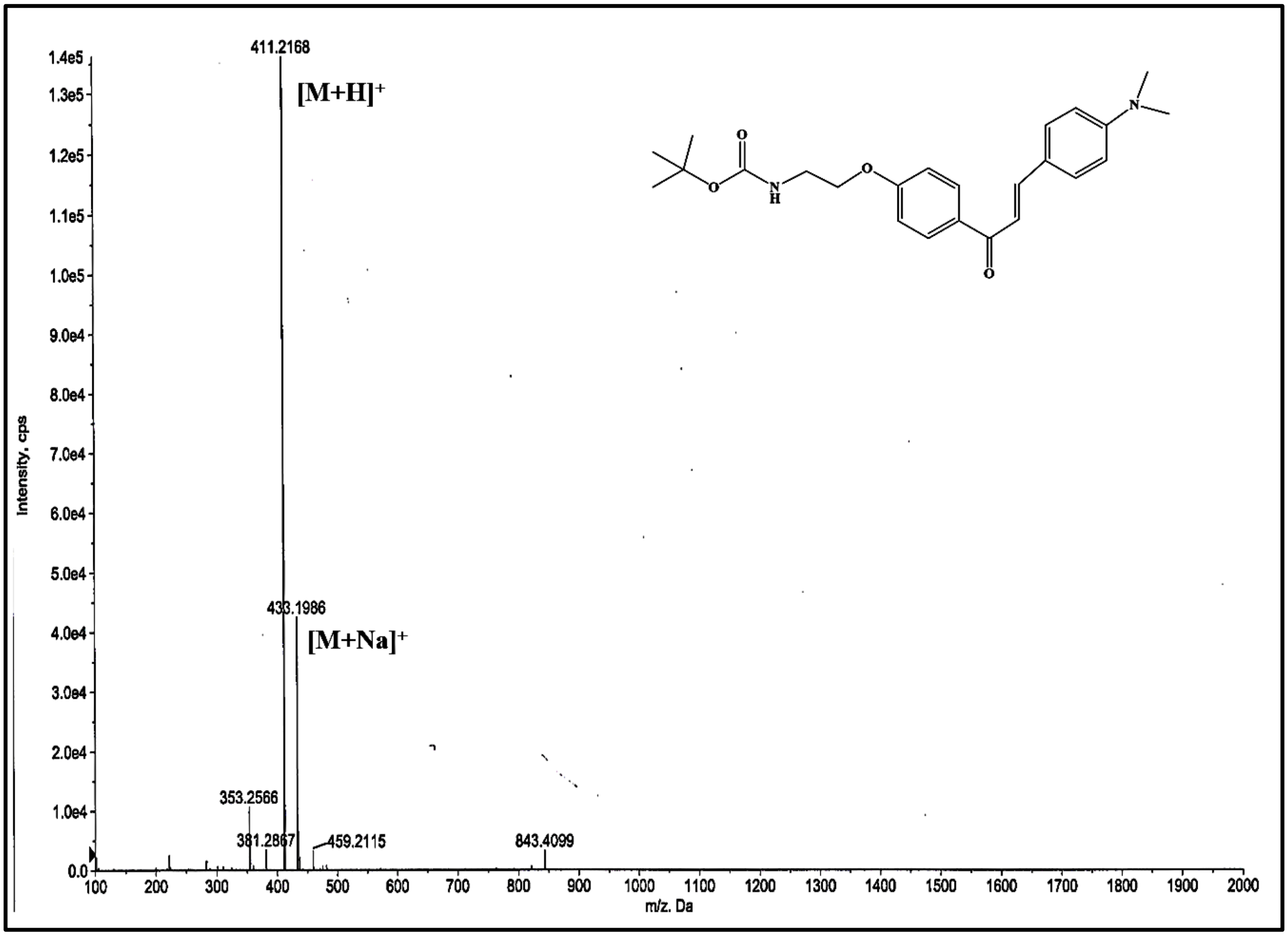

Figure S18. ESI-MS spectrum of (E)-*tert*-butyl 2-(4-(3-(4-(dimethylamino)phenyl)acryloyl)phenoxy)ethylcarbamate (3)

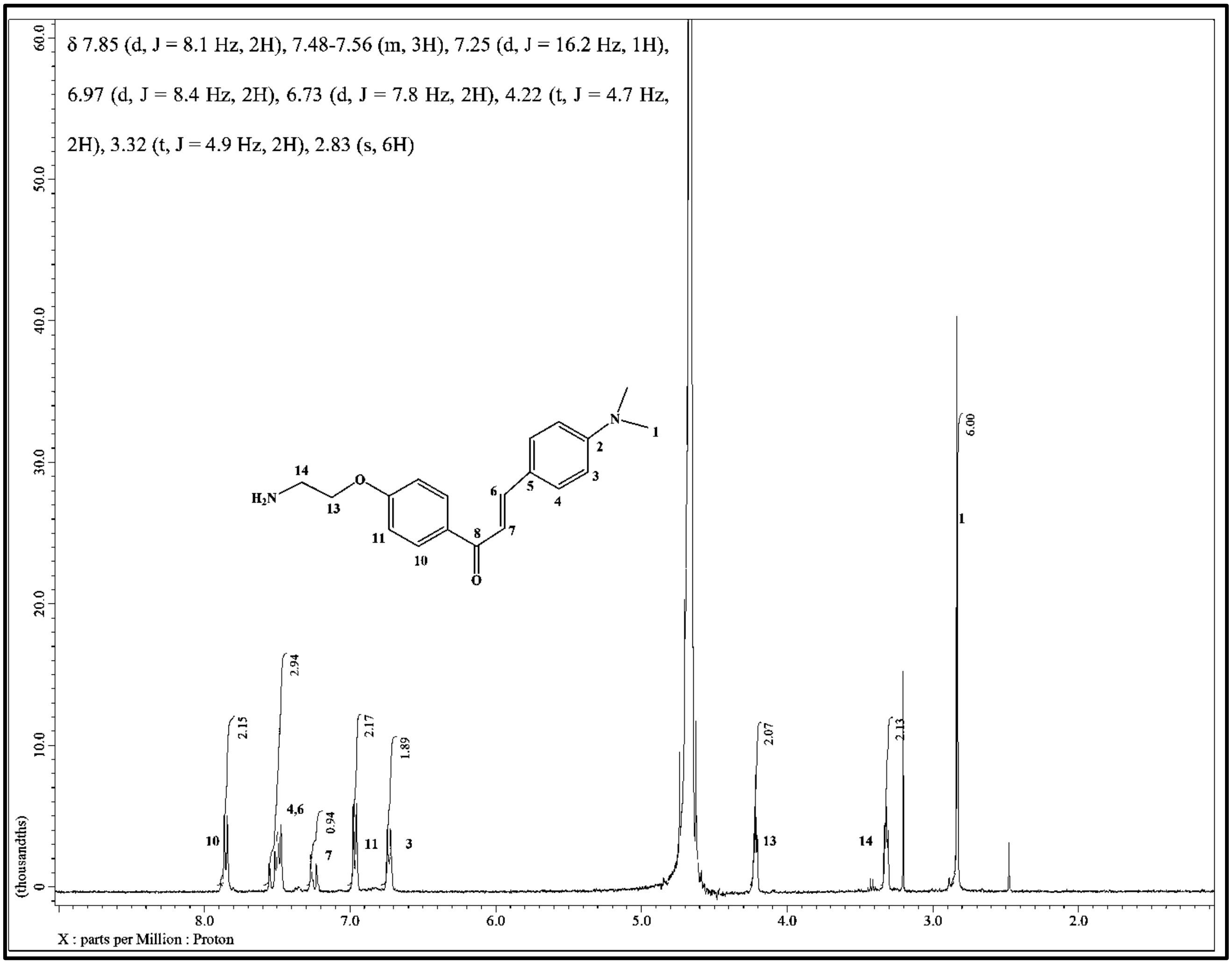

Figure S19. ^{1}H NMR spectrum of (E)-1-(4-(2-aminoethoxy)phenyl)-3-(4-(dimethylamino)phenyl)prop-2-en-1-one (4)

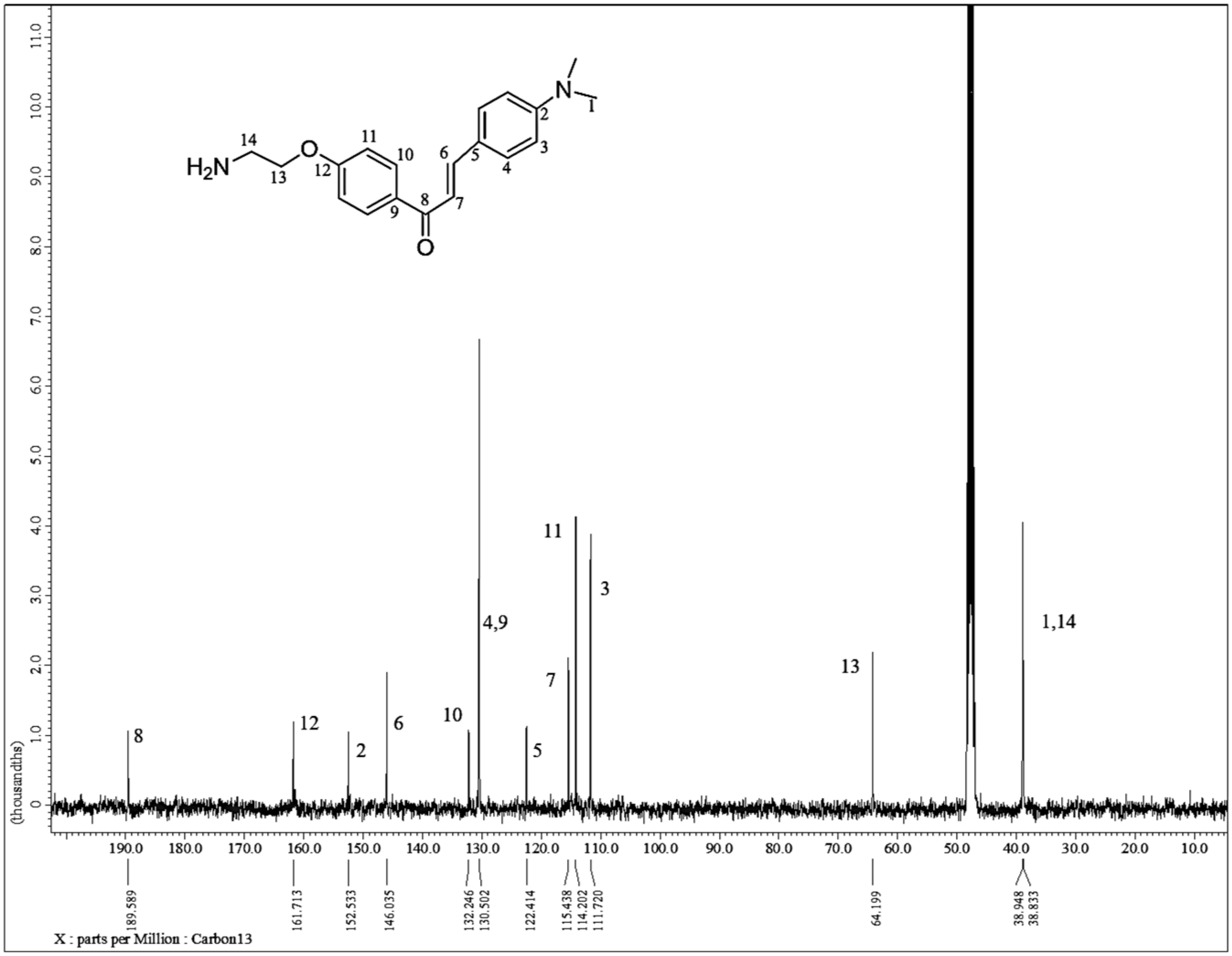

Figure S20. ^{13}C NMR spectrum of (E)-1-(4-(2-aminoethoxy)phenyl)-3-(4-(dimethylamino)phenyl)prop-2-en-1-one (4)

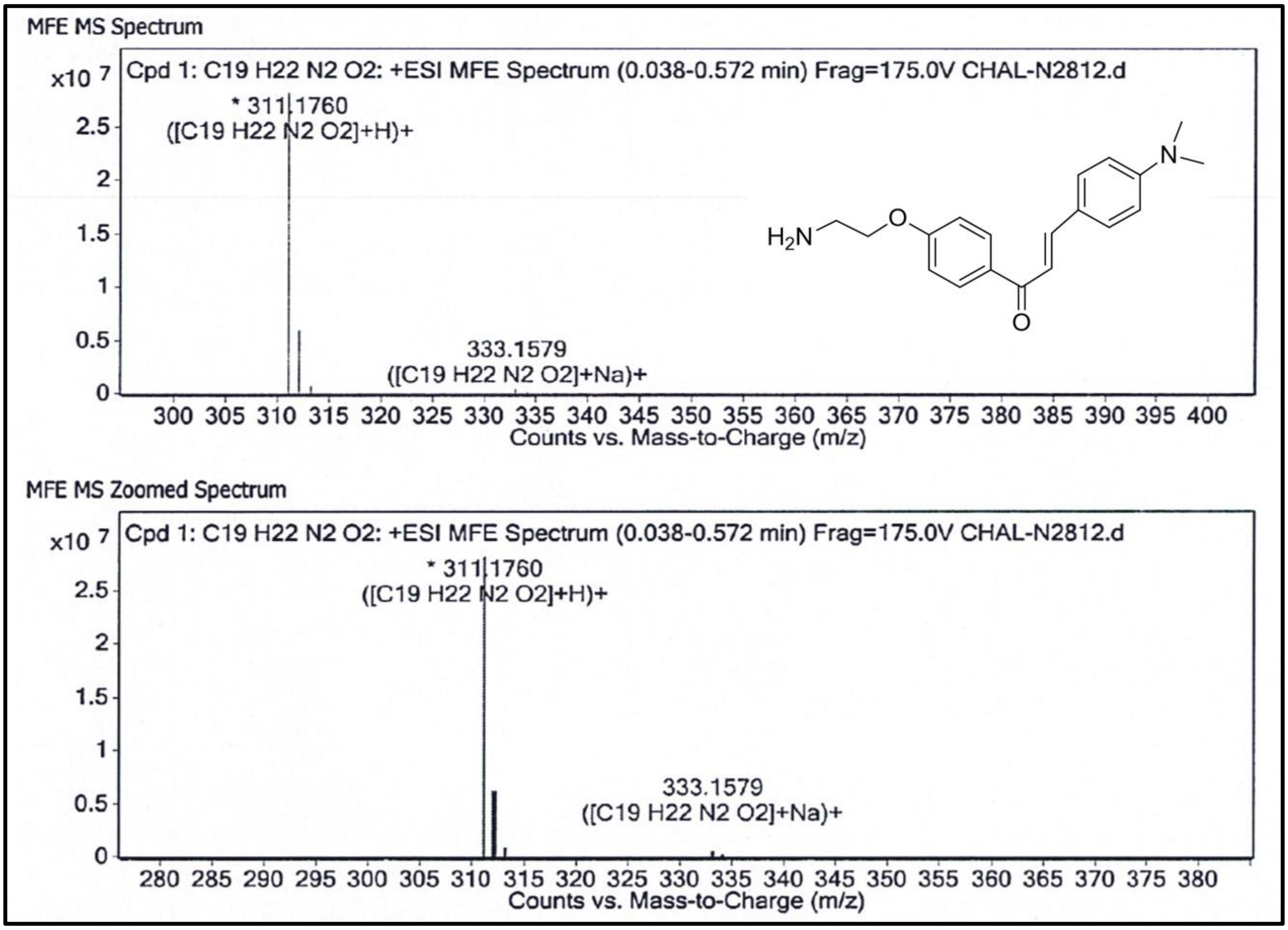

Figure S21. ESI-MS spectrum of (E)-1-(4-(2-aminoethoxy)phenyl)-3-(4-(dimethylamino)phenyl)prop-2-en-1-one (4)

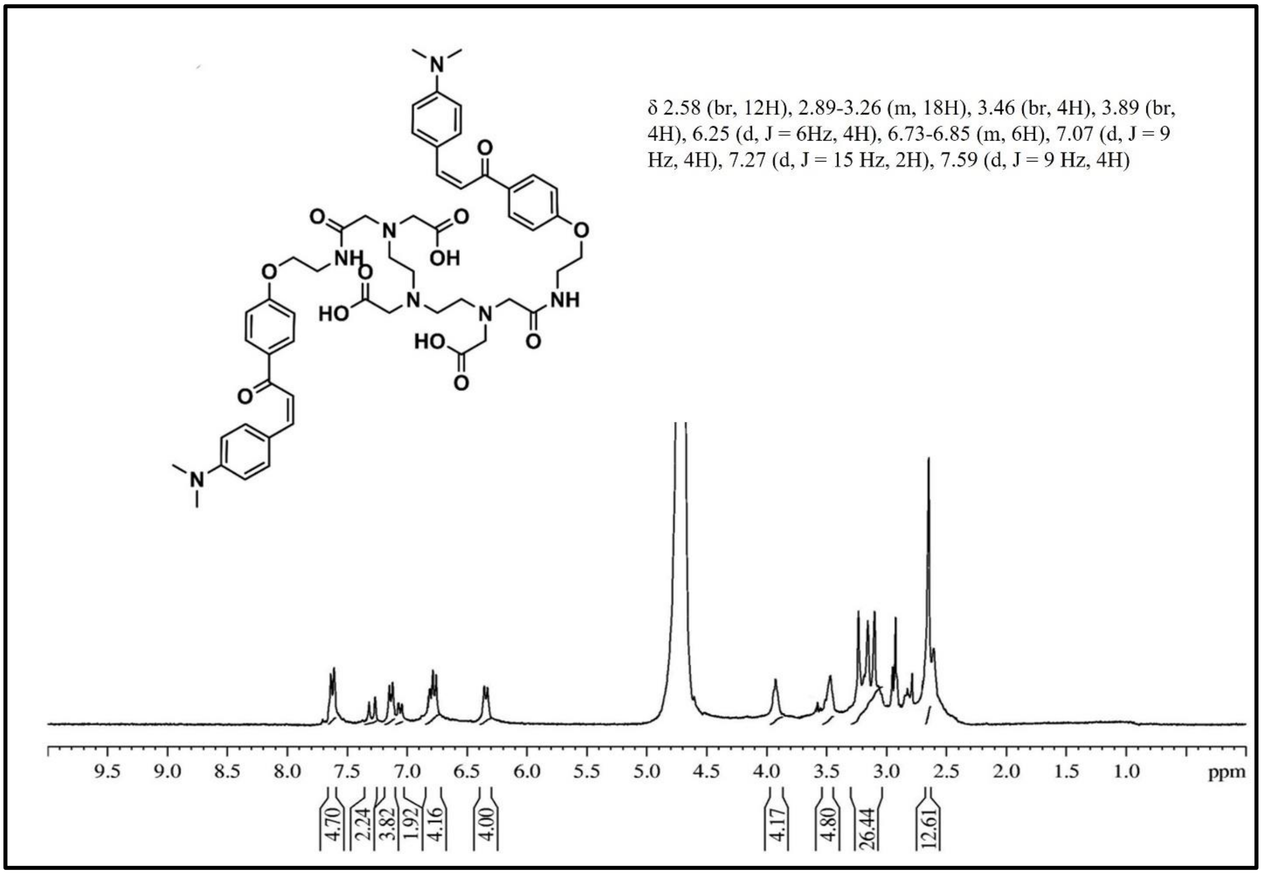

Figure S22. ^{1}H NMR spectrum of 6,9-bis(carboxymethyl)-14-(4-((E)-3-(4-(dimethylamino)phenyl)acryloyl)phenoxy)-3-(2-((2-(4-((E)-3-(4-(dimethylamino)phenyl)acryloyl)phenoxy)ethyl)amino)-2-oxoethyl)-11-oxo-3,6,9,12-tetraazatetradecanoic acid (5)

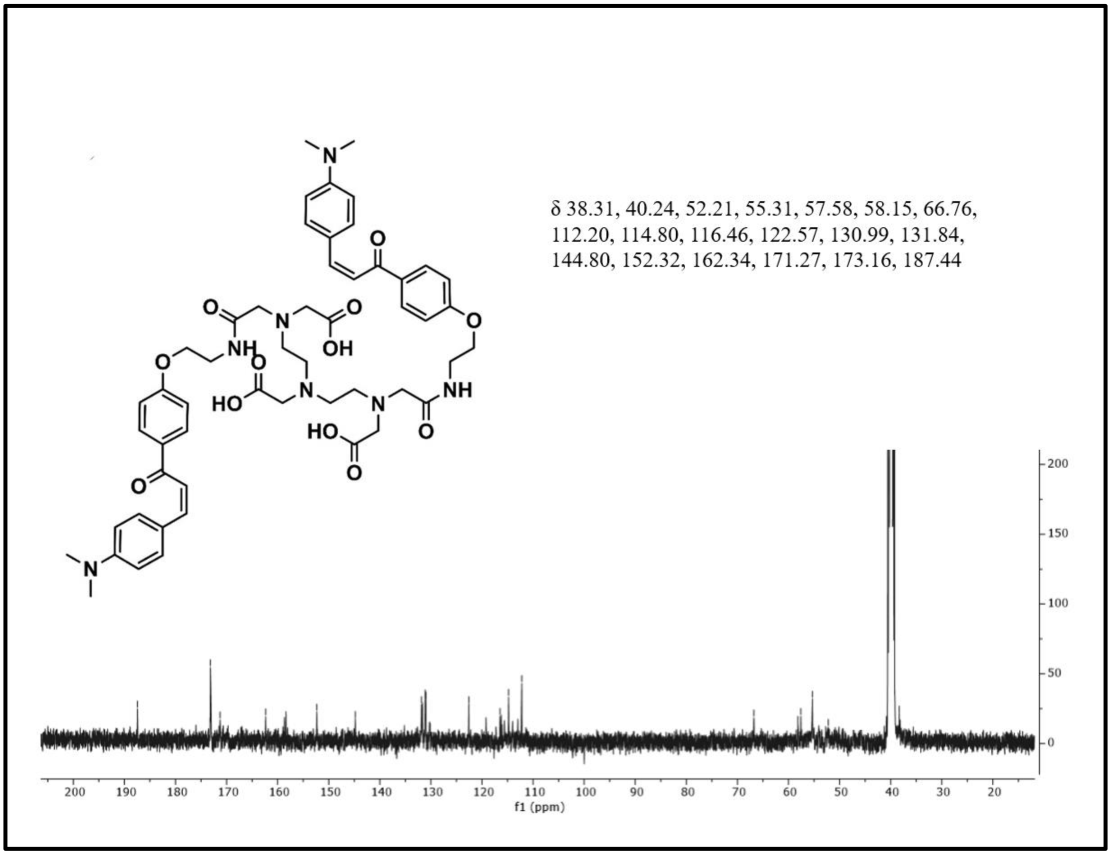

Figure S23. ^{13}C NMR spectrum of 6,9-bis(carboxymethyl)-14-(4-((E)-3-(4-(dimethylamino)phenyl)acryloyl)phenoxy)-3-(2-((2-(4-((E)-3-(4-(dimethylamino)phenyl)acryloyl)phenoxy)ethyl)amino)-2-oxoethyl)-11-oxo-3,6,9,12-tetraazatetradecanoic acid (5)

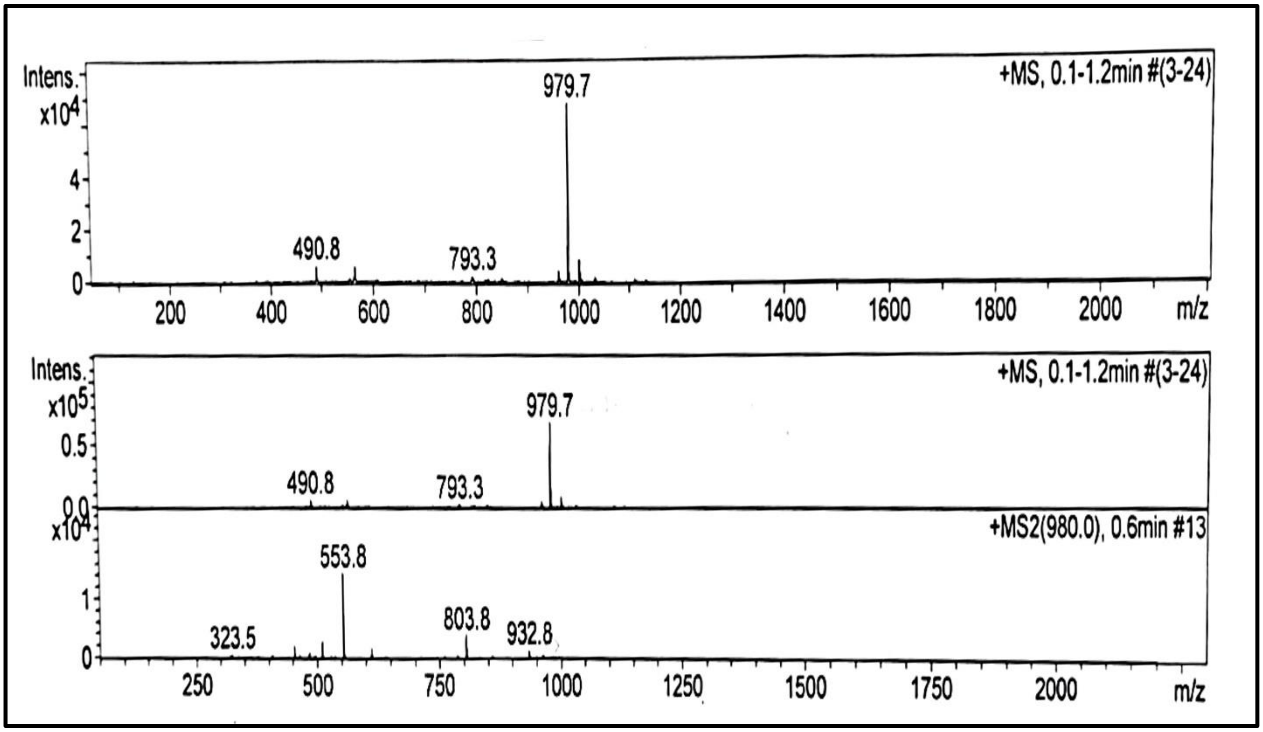

Figure S24. ESI-MS spectrum of 6,9-bis(carboxymethyl)-14-(4-((E)-3-(4-(dimethylamino)phenyl)acryloyl)phenoxy)-3-(2-((2-(4-((E)-3-(4-(dimethylamino)phenyl)acryloyl)phenoxy)ethyl)amino)-2-oxoethyl)-11-oxo-3,6,9,12-tetraazatetradecanoic acid (5)

References

1. Chauhan K, Datta A, Adhikari A, Chuttani K, Kumar Singh A, Mishra AK. (68)Ga Based Probe for Alzheimer's Disease: Synthesis and Preclinical Evaluation of Homodimeric Chalcone in Beta-Amyloid Imaging. *Org Biomol Chem* (2014) 12(37):7328-37. Epub 2014/08/15. doi: 10.1039/c4ob00941j.
